# Supplementary material for: An epitranscriptomic program maintains skeletal stem cell quiescence via a METTL3-FEM1B-GLI1 axis
Source: EMBO J. 2025 Feb 27;44(8):2263–78. doi: 10.1038/s44318-025-00399-z (PMC12000498; doi:10.1038/s44318-025-00399-z)
Supplement: Supplementary file 1 — Appendix [file 44318_2025_399_MOESM1_ESM.pdf]

# Appendix

## An epitranscriptomic program maintains skeletal stem cell quiescence via a METTL3-FEM1B-GLI1 axis

Jing Wang, Weidong Liu, Tiantian Zhang, Manman Cui, Kexin Gao, Pengbo Lu, Shuxin Yao, Ziyan Cao, Yanbing Zheng, Wen Tian, Yan Li, Rong Yin, Jin Hu, Guoqiang Han, Jianfei Liang, Fuling Zhou, Jihua Chai, Haojian Zhang

**Table of Contents**

**Appendix Figure S1.** Comprehensive m$^6$A landscape across skeletal hierarchy (pages 2-3).

**Appendix Figure S2.** Mettl3 deficiency causes bone dysplasia (pages 4-5).

**Appendix Figure S3.** Loss of Mettl3 impairs the quiescence of SSCs (pages 6).

**Appendix Figure S4.** Loss of Mettl3 impairs the multipotency of SSCs (pages 7-8).

**Appendix Figure S5.** Mettl3 regulates Fem1b stability to maintain SSCs function (pages 9).

**Appendix Figure S6.** Gli1 mediates the function of Mettl3 in maintaining the function of SSCs (pages 10-11).

**Appendix Table S1.** List of Primer sequences (Related to Reagents and Tools Table) (pages 12-13).

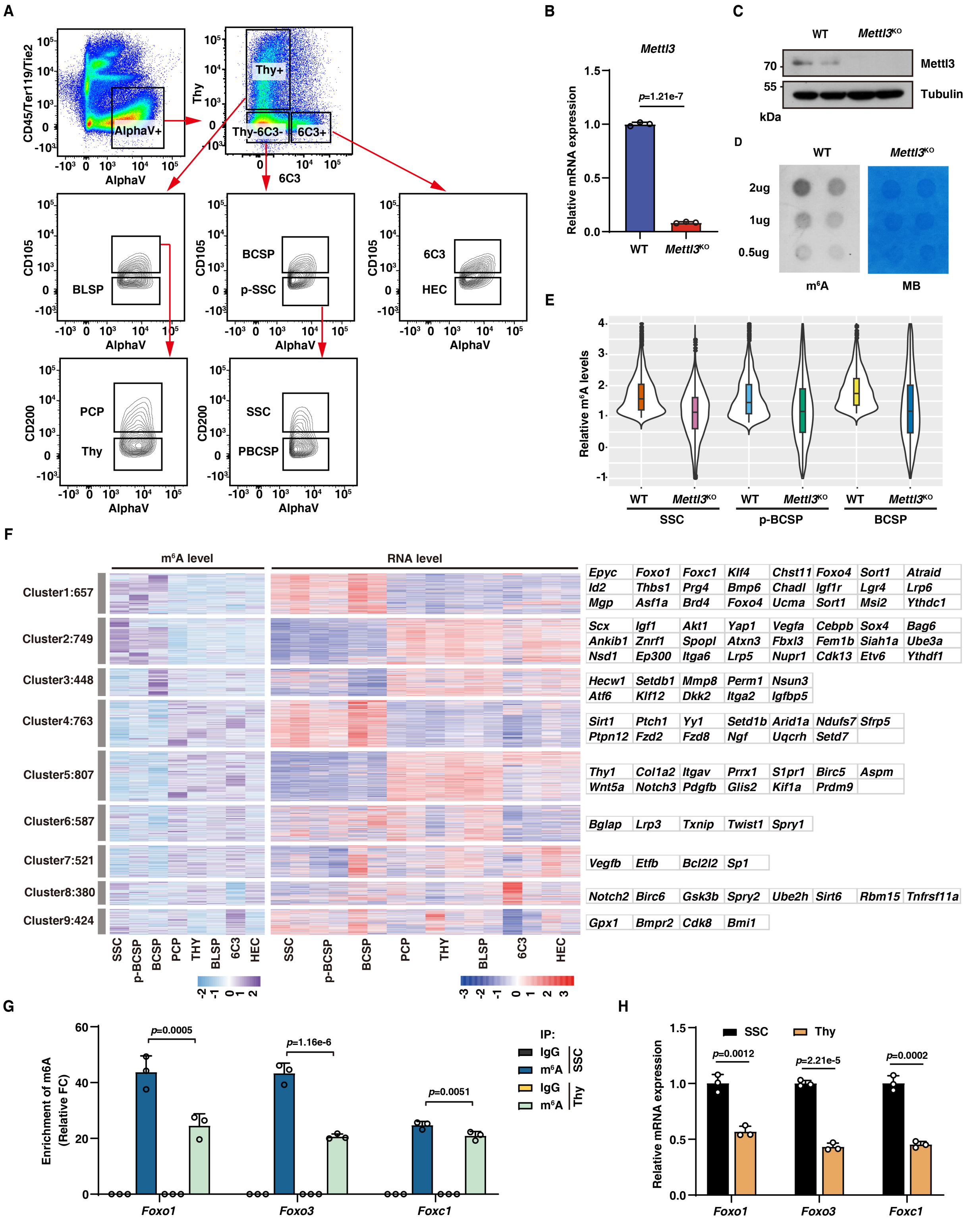


**Appendix Figure S1. Comprehensive m$^6$A landscape across skeletal hierarchy.**

A. Schematic diagram of gating strategy of mouse skeletal stem cells.

B. qRT-PCR analysis showing knockout efficiency of *Mettl3* deletion. n = 3, n represents biological independent experiments.

C. Western blot assay showing Mettl3 expression in SSCs from WT mice and *Mettl3*$^{KO}$ mice. Tubulin served as the loading control.

D. The m$^6$A dot blot showing m$^6$A level of total RNA of stem-progenitors from WT mice and *Mettl3*$^{KO}$ mice upon Mettl3 loss. MB, methylene blue staining (as loading control).

E. Violin plots showing decreased $m^6A$ levels of SSCs, P-BCSP, BCSP upon Mettl3 loss. n = 2, n represents biological independent experiments. Data were presented as box-and-whisker plots (medians with interquartile range, and the whiskers extend to the smallest and largest data points within 1.5 times the IQR).
F. Comprehensive correlation and GO enrichment analysis of $m^6A$ and RNA levels in different populations. Heatmap showing 5327 $m^6A$-tagged targets clustered with K-mean(K=9), and clusters (C1-C9) were shown. Left is $m^6A$ and mRNA expression levels, right is important lineage-specific genes.
G. MeRIP-PCR showing $m^6A$ levels of indicated master regulators in SSCs and the Thy-subtype.
H. RT-qPCR showing mRNA levels of indicated master regulators in SSCs and the Thy-subtype.

Data information: The data in B, H was analyzed by unpaired two-tailed student's t-test. The data in G were analyzed by one-way ANOVA with Dunnett's multiple comparisons test. The error bars represent mean ± S.D.

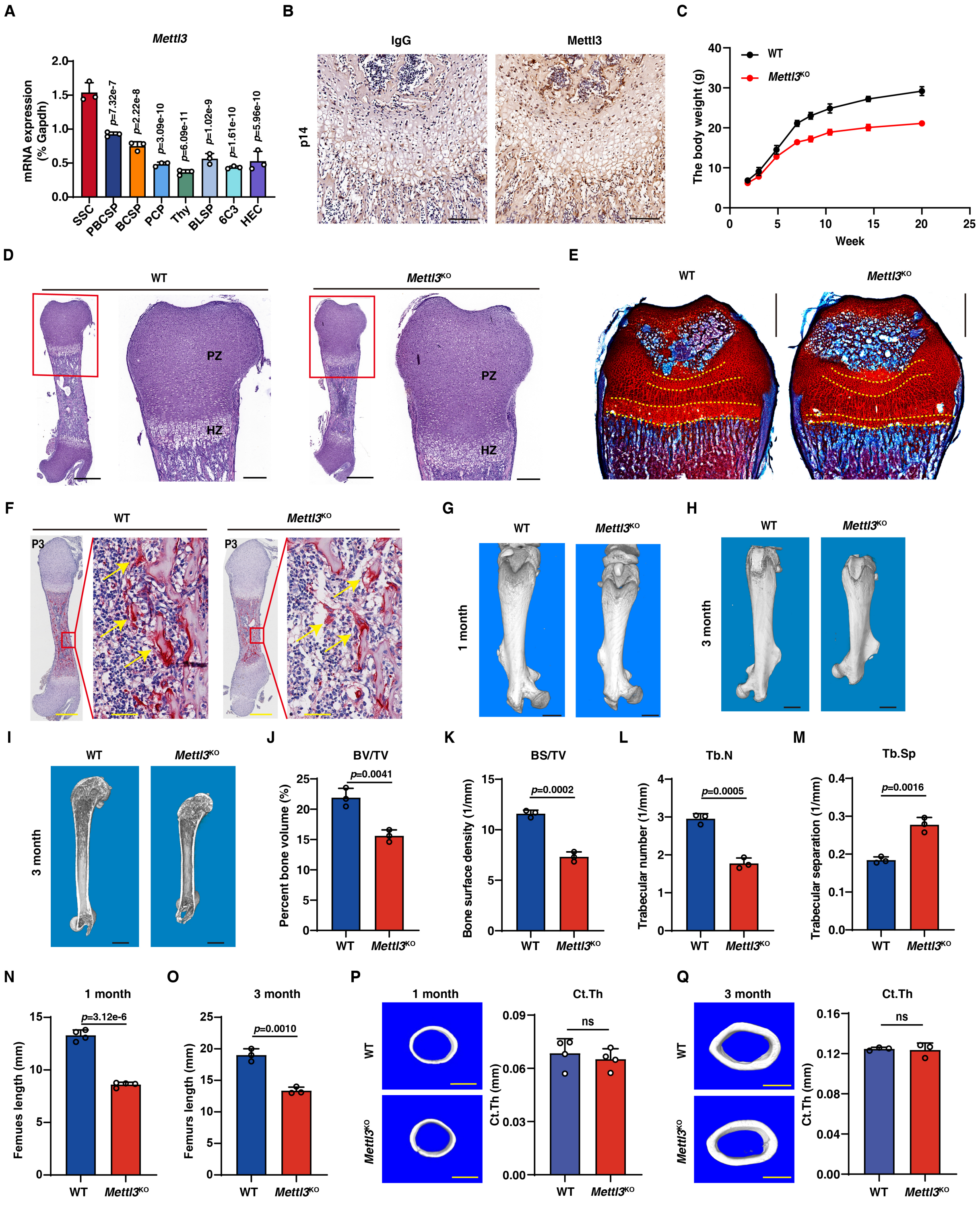


**Appendix Figure S2. Mettl3 deficiency causes bone dysplasia.**

A. Expression levels of *Mettl3* in the indicated stem and progenitor cells. *Gapdh* was used as an internal control. n = 3, n represents biological independent experiments.
B. Immunohistochemistry analysis of Mettl3 expression in the growth plate of femurs at P14. Scale bars, 100 μm.
C. Quantification of body weight of WT mice and *Mettl3*$^{KO}$ mice at different ages. n = 5.
D. H&E staining showing the height of growth plates in femurs at P3. Scale bars, the left is 500 μm, the right is 200 μm.

E. Safranin O-fast green staining showing the cartilage area in femurs at P14. Scale bars, 500 μm.
F. TRAP staining of femurs sections at P3. Arrowheads, osteoclast. Scale bars, the left is 500 μm, the right is 50 μm.
G. Micro-CT analysis showing the three-dimensional reconstruction images of femurs at 1 month. Scale bar, 1 mm.
H. Micro-CT analysis showing the three-dimensional reconstruction images of femurs at 3 month. Scale bar, 2 mm.
I. Micro-CT analysis showing the three-dimensional reconstruction images of cancellous bones of femurs at 3 month. Scale bar, 2 mm.
J-M. Quantification measurements of the bone volume/total volume (BV/TV), bone surface/total volume (BS/TV), trabecular number (Tb.N), and trabecular separation (Tb.Sp) of femurs from 3-month-old WT mice and $Mettl3^{KO}$ mice (n = 3) by micro-CT.
N. Quantification of femurs length of WT mice and $Mettl3^{KO}$ mice at 1 month. n = 4.
O. Quantification of femurs length of WT mice and $Mettl3^{KO}$ mice at 3 month. n = 3.
P. The left showing the photomicrographs of representative μCT-3D scan of femurs at 1 month, the right is the quantification measurements of the Ct thickness (Ct.Th). Scale bar, 1 mm. n = 4.
Q. The left showing the photomicrographs of representative μCT-3D scan of femurs at 3 month, the right is the quantification measurements of the Ct thickness (Ct.Th). Scale bar, 1 mm. n = 3.

Data information: The data in A were analyzed by one-way ANOVA with Dunnett's multiple comparisons test. The data in J-Q was analyzed by unpaired two-tailed student's t-test. The error bars represent mean ± S.D; ns, no significance.

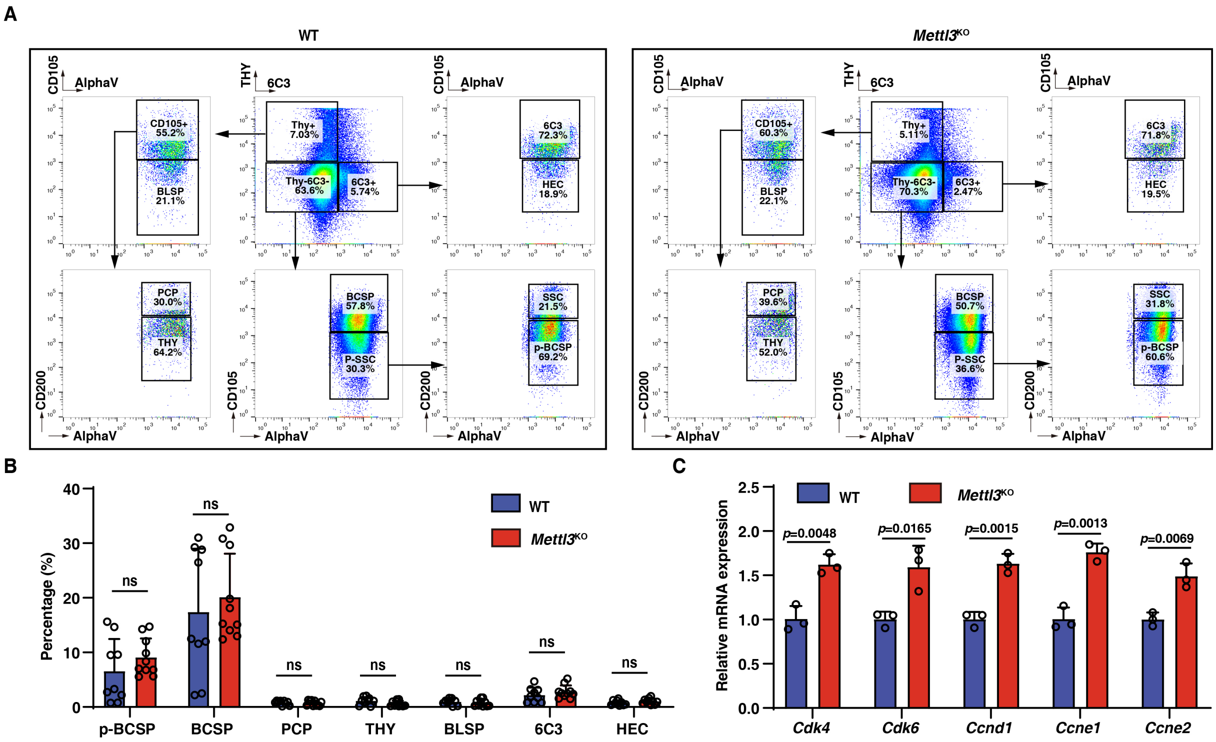


**Appendix Figure S3. Loss of Mettl3 impairs the quiescence of SSCs.**

A. Gated cell populations in flow cytometry of SSCs, p-BCSPs, BCSPs, PCPs, BLSPs, THYs, 6C3s and HECs in femurs of P3 WT mice and *Mettl3*$^{KO}$ mice.

B. Quantification of the SSCs progenies percentages identifies by surface markers. n = 9 or 10.

C. qRT-PCR analysis showing mRNA expression levels of cell cycle related genes of SSCs in WT mice and *Mettl3*$^{KO}$ mice. n = 3, n represents biological independent experiments.

Data information: The data in B-C was analyzed by unpaired two-tailed student's t-test. The error bars represent mean ± S.D; ns, no significance.

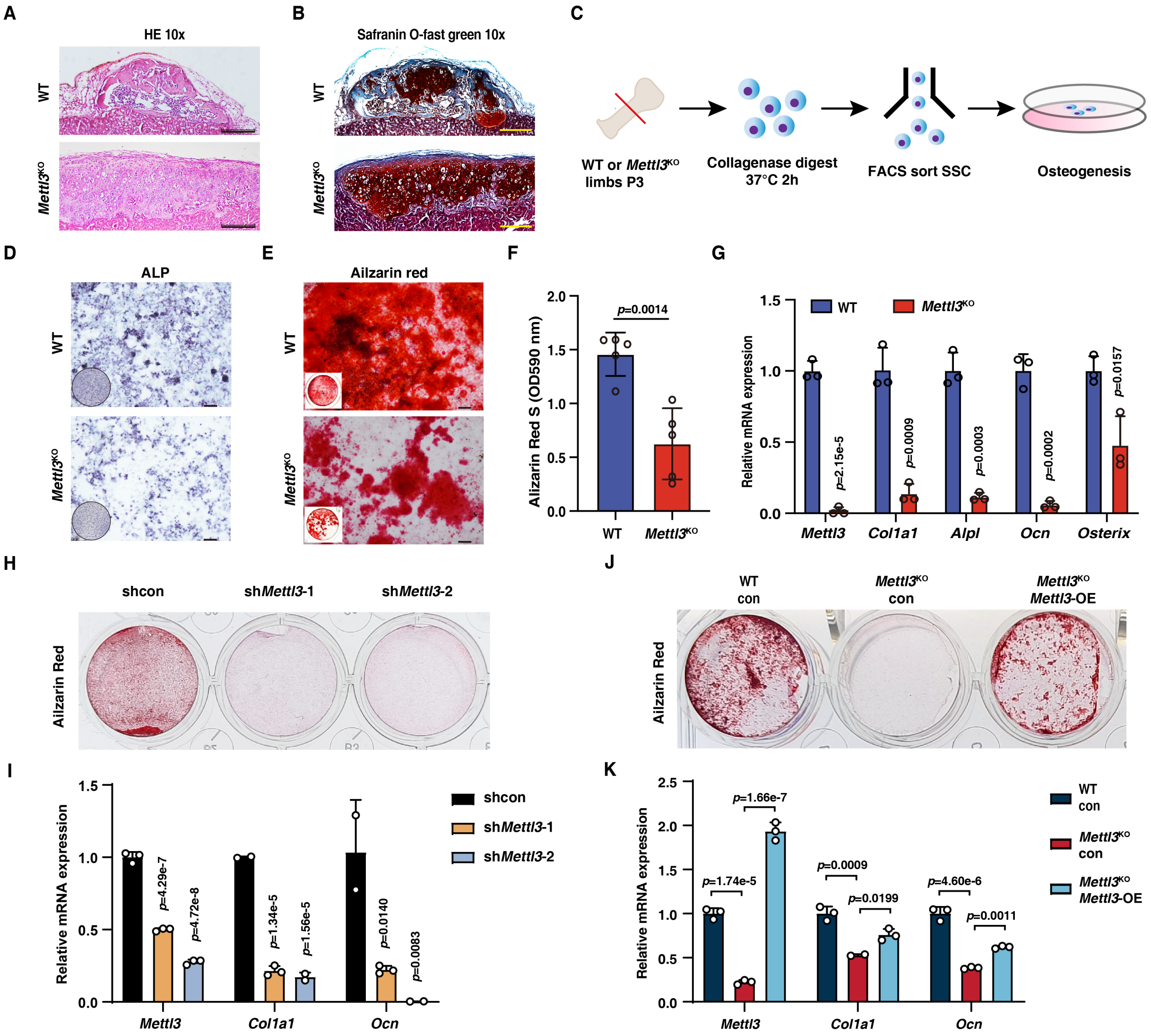


**Appendix Figure S4. Loss of Mettl3 impairs the multipotency of SSCs.**

A. H&E staining of tissue grafts following cell transplant beneath the renal capsule. Its cropped image was exhibited in Figure 3I. Scale bar, 200 μm.
B. Safranin O-fast green staining of tissue grafts following cell transplant beneath the renal capsule. Its cropped image was exhibited in Figure 3J. Scale bar, 200 μm.
C. Schematic diagram showing the functional analysis of SSC *ex vivo*.
D. Osteogenic differentiation of SSCs for 21 d stained with ALP. Representative images are shown (n = 3 per genotype). Scale bar, 200 μm.
E. Osteogenic differentiation of SSCs for 21 d stained with alizarin red. Representative images are shown (n = 3 per genotype). Scale bar, 200 μm.
F. Quantitative analysis of alizarin red staining. n = 5, n represents biological independent experiments.
G. qRT-PCR analysis of mRNA expression levels of osteogenic genes in SSCs. n = 3, n represents biological independent experiments.
H. Representative images of alizarin red staining in SSCs after knocking down Mettl3 on the 21 d of osteogenic induction. n = 3 per genotype.
I. qRT-PCR analysis of the expression of *Mettl3*, *Col1a1* and *Ocn* under osteogenic conditions. n = 3, n represents biological independent experiments.

J. Representative images of alizarin red staining in SSCs after overexpressing Mettl3 on the 21 d of osteogenic induction. n = 3 per genotype.
K. qRT-PCR analysis of the expression of *Mettl3*, *Col1a1* and *Ocn* under osteogenic conditions. n = 3, n represents biological independent experiments.

Data information: The data in F-G was analyzed by unpaired two-tailed student's t-test. The data in I, K were analyzed by one-way ANOVA with Dunnett's multiple comparisons test. The error bars represent mean ± S.D.

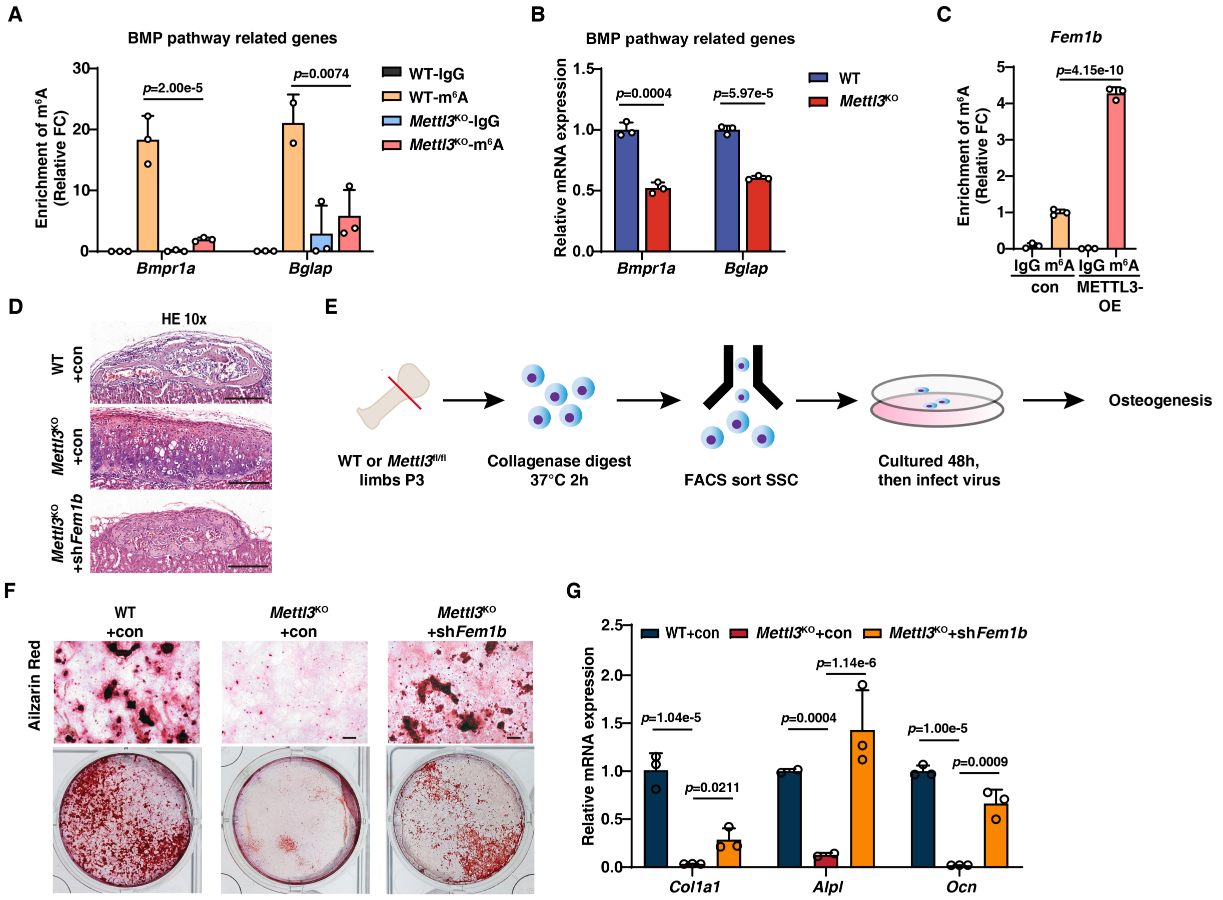


**Appendix Figure S5. Mettl3 regulates Fem1b stability to maintain SSCs function.**

A. MeRIP-PCR analysis of m$^6$A enrichment of mRNAs for *Bmpr1a* and *Bglap* in WT SSCs and *Mettl3*$^{KO}$ SSCs. n = 3, n represents biological independent experiments.
B. qRT-PCR analysis showing *Bmpr1a* and *Bglap* mRNA expression in WT SSCs and *Mettl3*$^{KO}$ SSCs. n = 3, n represents biological independent experiments.
C. MeRIP-PCR analysis of m$^6$A enrichment of mRNAs for *Fem1b* after altering *Mettl3* expression. n = 3, n represents biological independent experiments.
D. H&E staining of tissue grafts following cell transplant beneath the renal capsule. Its cropped image was exhibited in Figure 4M. Scale bar, 200 μm.
E. Schematic diagram showing the functional rescue Mettl3-deficient of SSCs *ex vivo*.
F. Alizarin red staining of osteogenic differentiation for 21 d of WT SSCs and *Mettl3*$^{KO}$ SSCs transduced with shcontrol or sh*Fem1b*. Representative images are shown (n = 3 per genotype). Scale bar, 200 μm.
G. qRT-PCR analysis of the expression of *Col1a1, Alpl,* and *Ocn* after infecting specific virus under osteogenic conditions. n = 3, n represents biological independent experiments.

Data information: The data in B was analyzed by unpaired two-tailed student's t-test. The data in A, C were analyzed by one-way ANOVA with Dunnett's multiple comparisons test. The data in G were analyzed by two-way ANOVA with Tukey's multiple comparisons test. The error bars represent mean ± S.D.

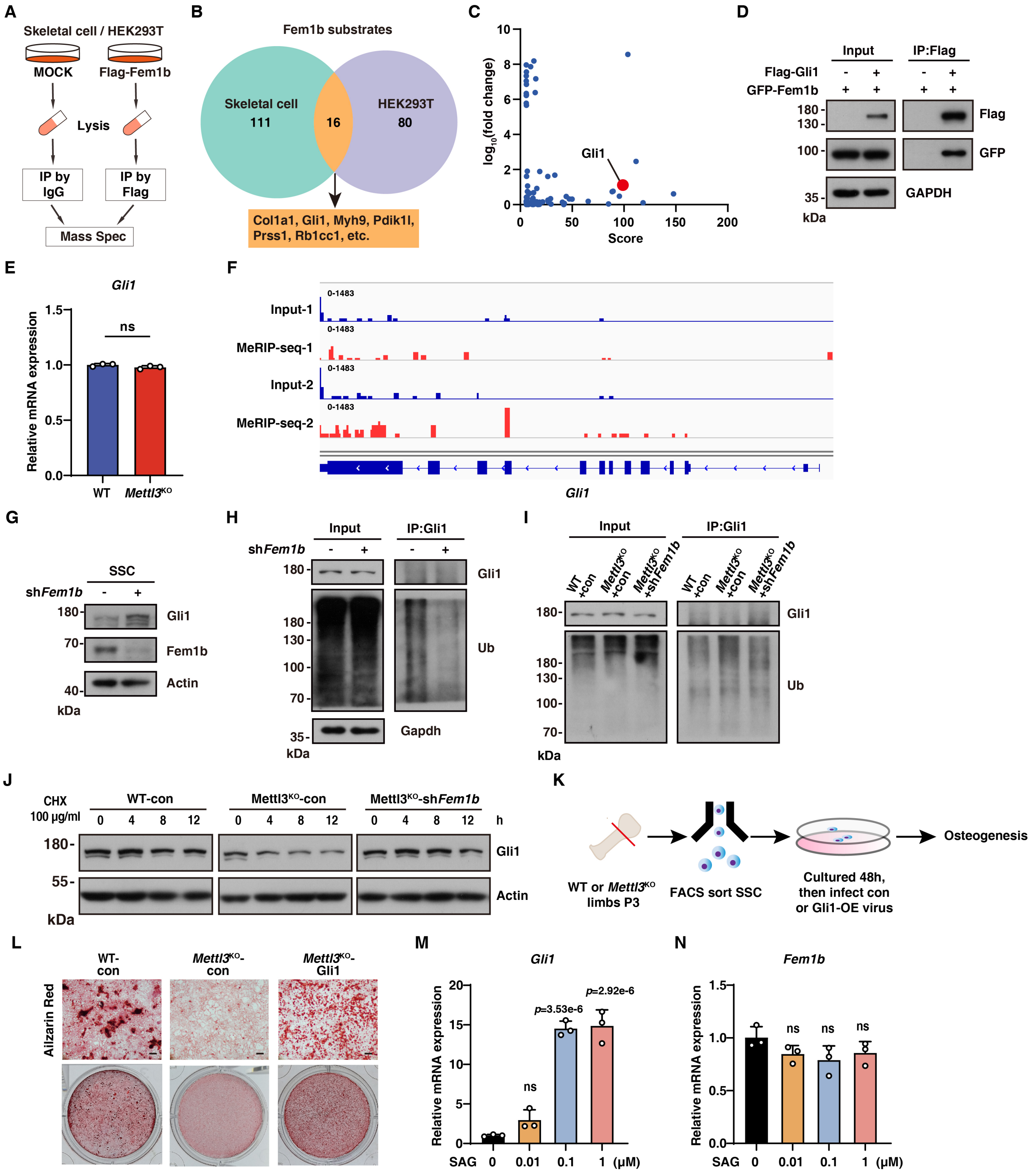


**Appendix Figure S6. Gli1 mediates the function of Mettl3 in maintaining the function of SSCs.**

A. Experimental scheme for Flag-Fem1b immunoprecipitation-mass spectrometry analysis in skeletal cells and HEK293T cells.
B. Venn diagrams showing the overlapped proteins pulled down by Flag-Fem1b protein in skeletal cells and HEK293T cells.
C. Mass spectrometry analysis of Fem1b interacting protein in skeletal cells. The top candidate was highlighted.
D. 293T cells were transfected with Flag-Gli1 and GFP-Fem1b, and cell lysates were co-immunoprecipitated with Anti-Flag Affinity Gel by immunoblotting analysis using the indicated antibodies.

E. qRT-PCR analysis showing *Gli1* mRNA expression in WT SSCs and *Mettl3*$^{KO}$ SSCs.
F. IGV tracks showing distribution m$^6$A peaks of Gli1transcripts in mesenchymal stem cells.
G. SSCs were transducted with retroviruses for shcontrol or sh*Fem1b*. Cellular extracts were prepared and analyzed by Western Blot.
H. Endogenous ubiquitination assay was analyzed in *Fem1b*-knockdown SSCs. Cell lysates were immunoprecipitated with Anti-Gli1 antibody and then analyzed by immunoblotting using the indicated antibodies.
I. Endogenous ubiquitination assay was analyzed in WT SSCs and *Mettl3*$^{KO}$ SSCs transduced with shcontrol or sh*Fem1b*.
J. WT SSCs and *Mettl3*$^{KO}$ SSCs transduced with shcontrol or sh*Fem1b* were treated with 100 µg/ml CHX, and harvested at the indicated time followed by Western blotting analysis.
K. Schematic diagram showing the functional rescue Mettl3-deficient of SSCs *ex vivo*.
L. Alizarin red staining of osteogenic differentiation for 21 d of WT SSCs and *Mettl3*$^{KO}$ SSCs transduced with control or Gli1-OE. Representative images are shown (n = 3 per genotype). Scale bar, 200 µm.
M. qRT-PCR analysis showing *Gli1* mRNA expression in SSCs treated with SAG.
N. qRT-PCR analysis showing *Fem1b* mRNA expression in SSCs treated with SAG.

Data information: The data in E was analyzed by unpaired two-tailed student's t-test. The data in M, N were analyzed by one-way ANOVA with Dunnett's multiple comparisons test. The error bars represent mean ± S.D; ns, no significance.

**Appendix Table S1. List of Primer sequences (Related to Reagents and Tools Table)**

| **Name** | **Sequence(5'-3')** | |
|---|---|---|
| **Qpcr** | **Forward** | **Reverse** |
| *Klf4* | GCCGGAGTTGGACCCAGTAT | CAGCACAAACTTGCCCATCAG |
| *Chst11* | GATGAACAGAATTTGCCGGATG | TGGAAATGCAGGTTCCTCATC |
| *Fem1b* | CTTTGCACTTTGCAGCCGAA | TCAATCCGACTTCTGCGGTC |
| *Mettl3* | CTGGGCACTTGGATTTAAGGAA | TGAGAGGTGGTGTAGCAACTT |
| *Sox9* | AGTACCCGCATCTGCACAAC | ACGAAGGGTCTCTTCTCGCT |
| *Acan* | CCTGCTACTTCATCGACCCC | AGATGCTGTTGACTCGAACCT |
| *Ihh* | CTCTTGCCTACAAGCAGTTCA | CCGTGTTCTCCTCGTCCTT |
| *Col10a1* | GGTGTGAATGGGCGGAAAG | GCTTCCCAATACCTTCTCGTC |
| *Gapdh* | AGGTCGGTGTGAACGGATTTG | GGGGTCGTTGATGGCAACA |
| *Actb* | GGGTTGGAAGACCGAGGTTTA | GCCAGGGTGACAATAATGGACA |
| *Col1a1* | TGGCAAAGACGGACTCAAC | GGCAGGAAGCTGAAGTCATAA |
| *Alpl* | CCAACTCTTTTGTGCCAGAGA | GGCTACATTGGTGTTGAGCTTTT |
| *Ocn* | AAGCAGGAGGGCAATAAGGT | TTTGTAGGCGGTCTTCAAGC |
| *Osterix* | CAACCTGCTAGAGATCTGAG | TGCAATAGGAGAGAGCGA |
| *Runx2* | ATTACAGATCCCAGGCAGGCA | CAGAAGTCAGAGGTGGCAGTGT |
| *Spopl* | GGAACGGCTGAAGGTCATGT | CACTGTGCAAATCTGCAAGGA |
| *Foxo1* | CGGAATGACCTCATGGATGGA | ACATGGTACTGTGTCCCTTTTT |
| *Foxo3* | CTCGGACTCTCTCTCAGGCT | CCCGTCAGCATCCATGAGTT |
| *Foxc1* | CCCCGGACAAGAAGATCACTC | AGGTTGTGCCGTATGCTGTTC |
| *Cdk4* | ATGGCTGCCACTCGATATGAA | TCCTCCATTAGGAACTCTCACAC |
| *Cdk6* | GGCGTACCCACAGAAACCATA | AGGTAAGGGCCATCTGAAAACT |
| *Ccnd1* | GCGTACCCTGACACCAATCTC | CTCCTCTTCGCACTTCTGCTC |
| *Ccne1* | GTGGCTCCGACCTTTCAGTC | CACAGTCTTGTCAATCTTGGCA |
| *Ccne2* | ATGTCAAGACGCAGCCGTTTA | GCTGATTCCTCCAGACAGTACA |
| **Genotyping** | **Forward** | **Reverse** |
| *Mettl3* | TCAGGATTTGCTCCTAGTGTATGCC | ACCAGGCCCTTGGAATACTTGGC |
| *Col2*-cre-wt | ATCGCCCAAATGACACCTCC | TCGCAATGGATTGTGTTGTTTC |
| *Col2*-cre-mut | ATCGCCCAAATGACACCTCC | TGGTGCATAGTCAGCAGGTTG |
| **shRNA** | | |
| sh*Mettl3*#1-F | CCGGCGTCAGTATCTTGGGCAAATTCTCGAGAATTTGCCCAAGATACTGACGTTTTTG | |
| sh*Mettl3*#1-R | AATTCAAAAACGTCAGTATCTTGGGCAAATTCTCGAGAATTTGCCCAAGATACTGACG | |
| shMettl3#2-F | CCGGGCACCCGCAAGATTGAGTTATCTCGAGATAACTCAATCTTGCGGGTGCTTTTTG | |

| | |
|---|---|
| shMettl3#2-R | AATTCAAAAAGCACCCGCAAGATTGAGTTATCTCGAGATAACTCAATCTTGCGGGTGC |
| shFem1b-F | TGCTGTTATATGCTGCGATCATTAGGGTTTTGGCCACTGACTGACCCTAATGAGCAGCATATAA |
| shFem1b-R | CCTGTTATATGCTGCTCATTAGGGTCAGTCAGTGGCCAAAACCCTAATGATCGCAGCATATAAC |
