# Supplementary figures and images for: An epitranscriptomic program maintains skeletal stem cell quiescence via a METTL3-FEM1B-GLI1 axis
Source: EMBO J. 2025 Feb 27;44(8):2263–78. doi: 10.1038/s44318-025-00399-z (PMC12000498; doi:10.1038/s44318-025-00399-z)

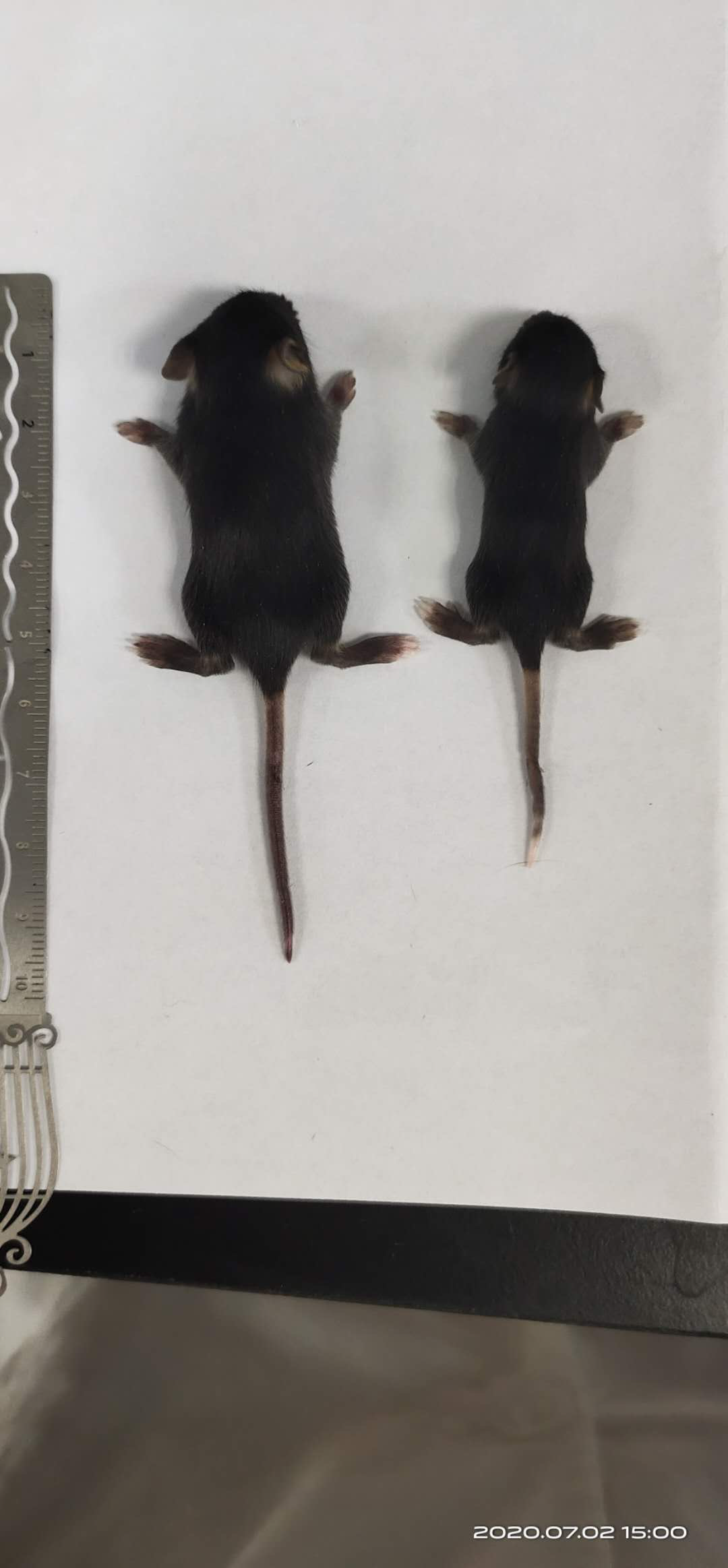

Supplement: Supplementary file 5 — Source data Fig. 2 [file 44318_2025_399_MOESM5_ESM.zip › Figure 2/2A/P14 mice.tif]

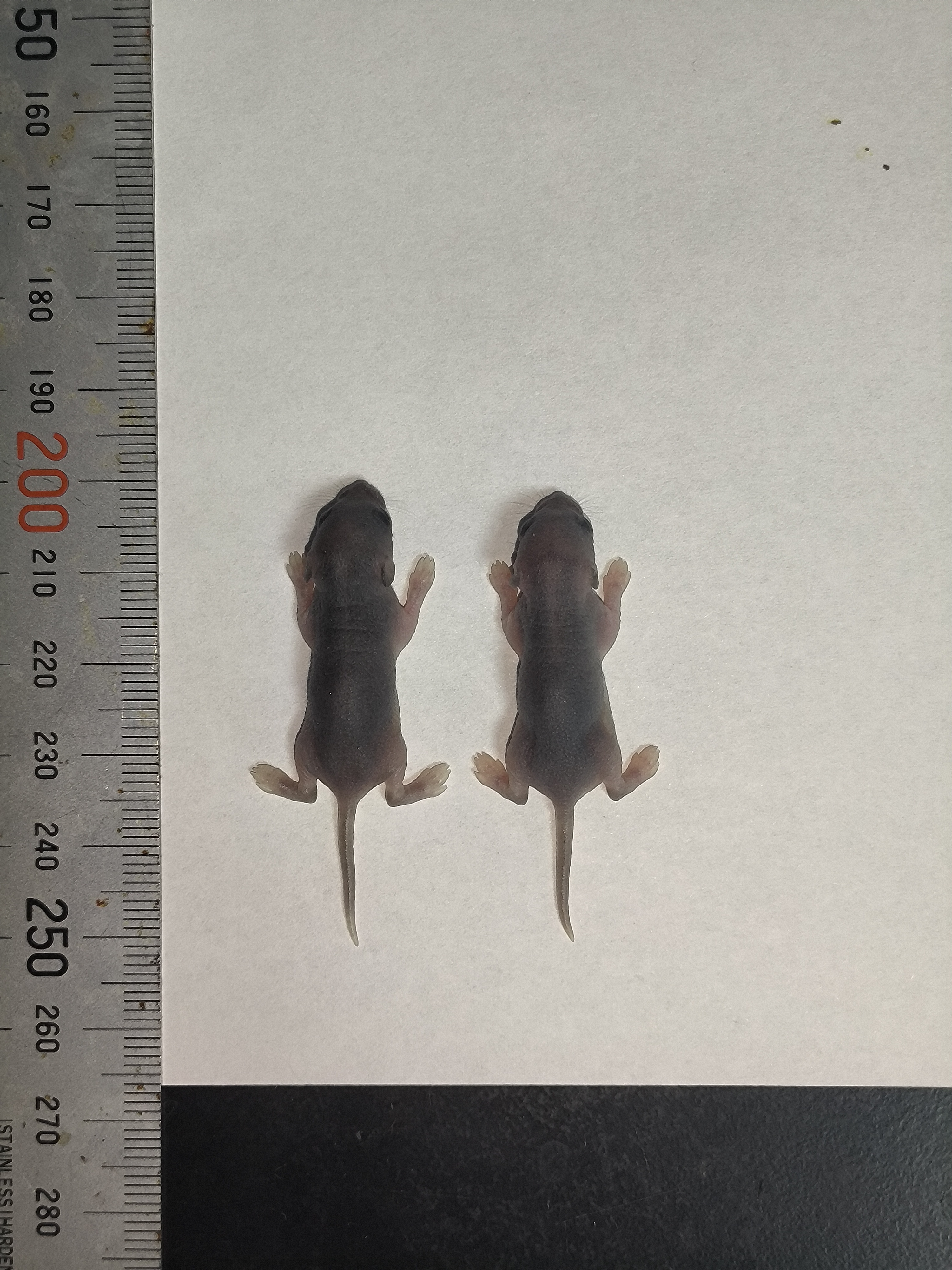

Supplement: Supplementary file 5 — Source data Fig. 2 [file 44318_2025_399_MOESM5_ESM.zip › Figure 2/2A/P3 mice.tif]

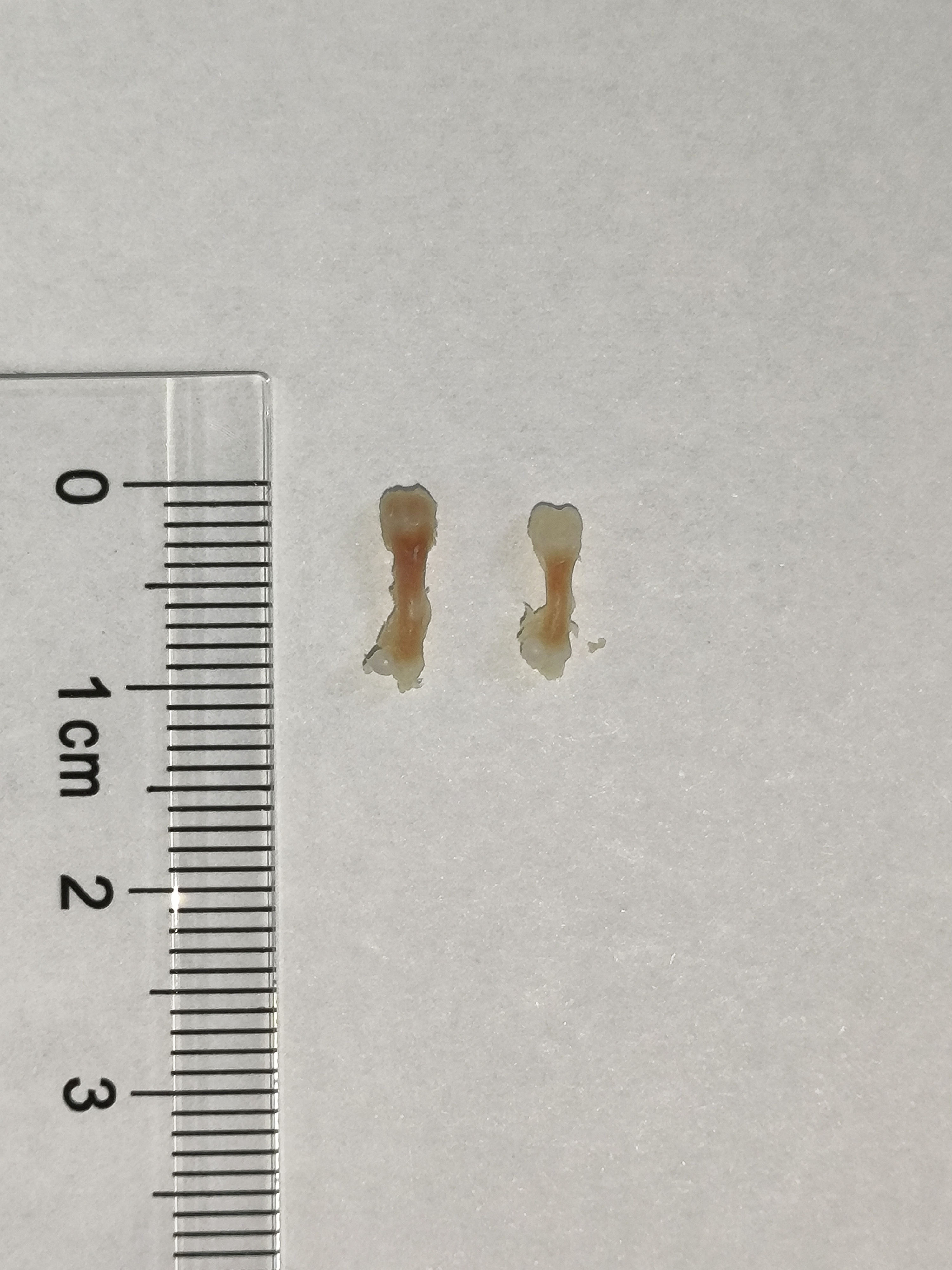

Supplement: Supplementary file 5 — Source data Fig. 2 [file 44318_2025_399_MOESM5_ESM.zip › Figure 2/2C/P14 femurs.tif]

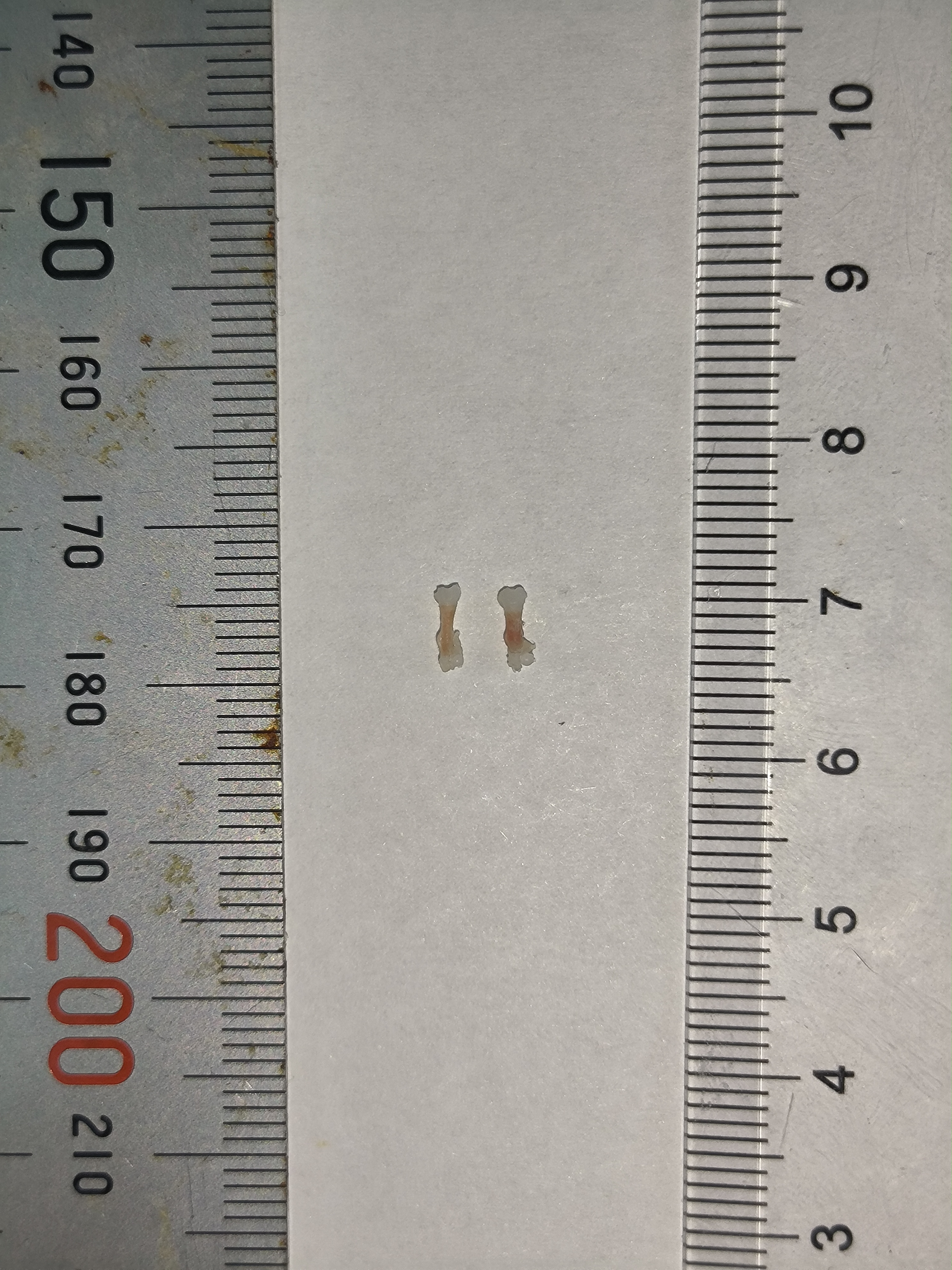

Supplement: Supplementary file 5 — Source data Fig. 2 [file 44318_2025_399_MOESM5_ESM.zip › Figure 2/2C/P3 femurs.tif]

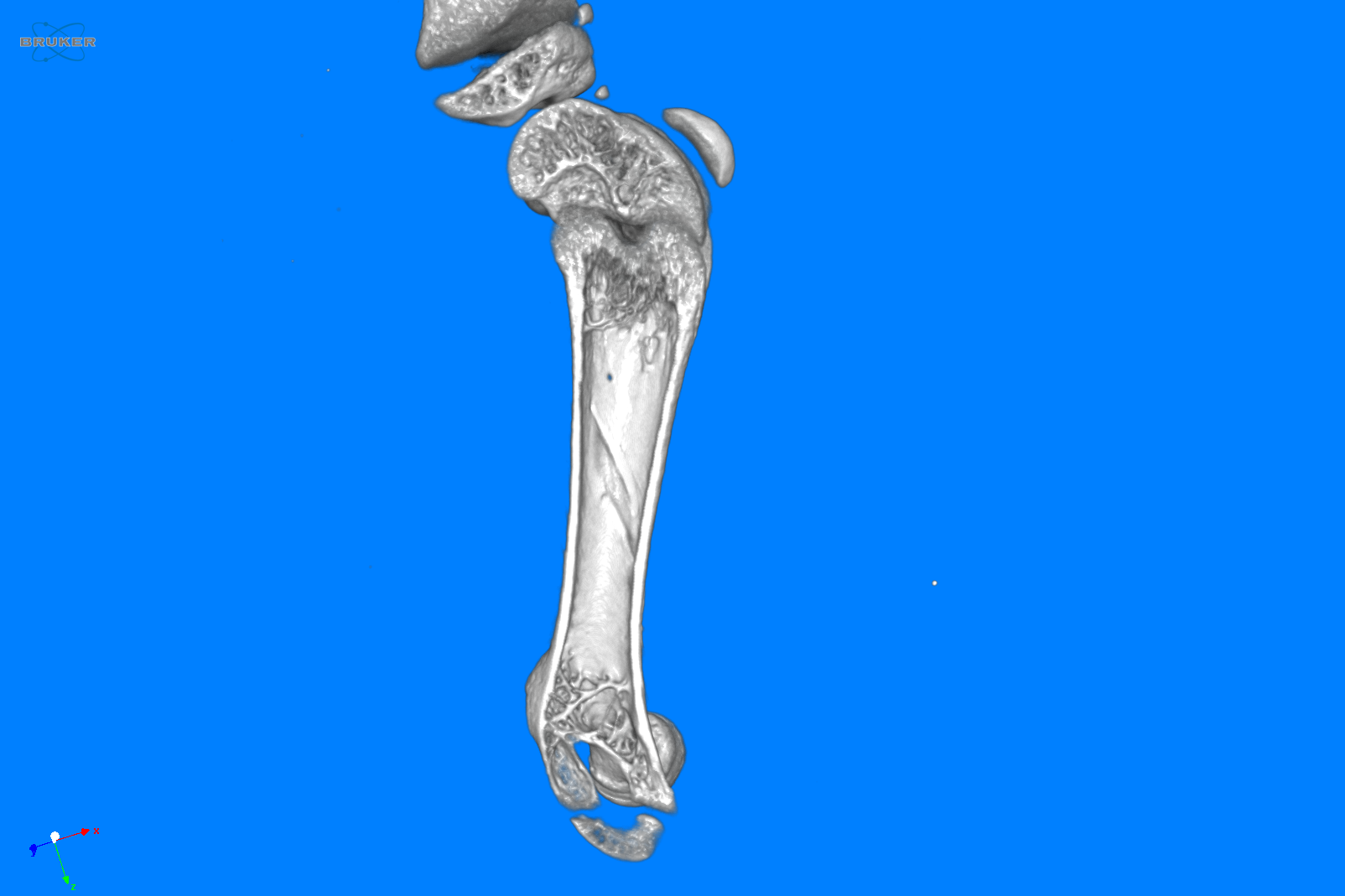

Supplement: Supplementary file 5 — Source data Fig. 2 [file 44318_2025_399_MOESM5_ESM.zip › Figure 2/2J/Fig2J-Mettl3KO.tif]

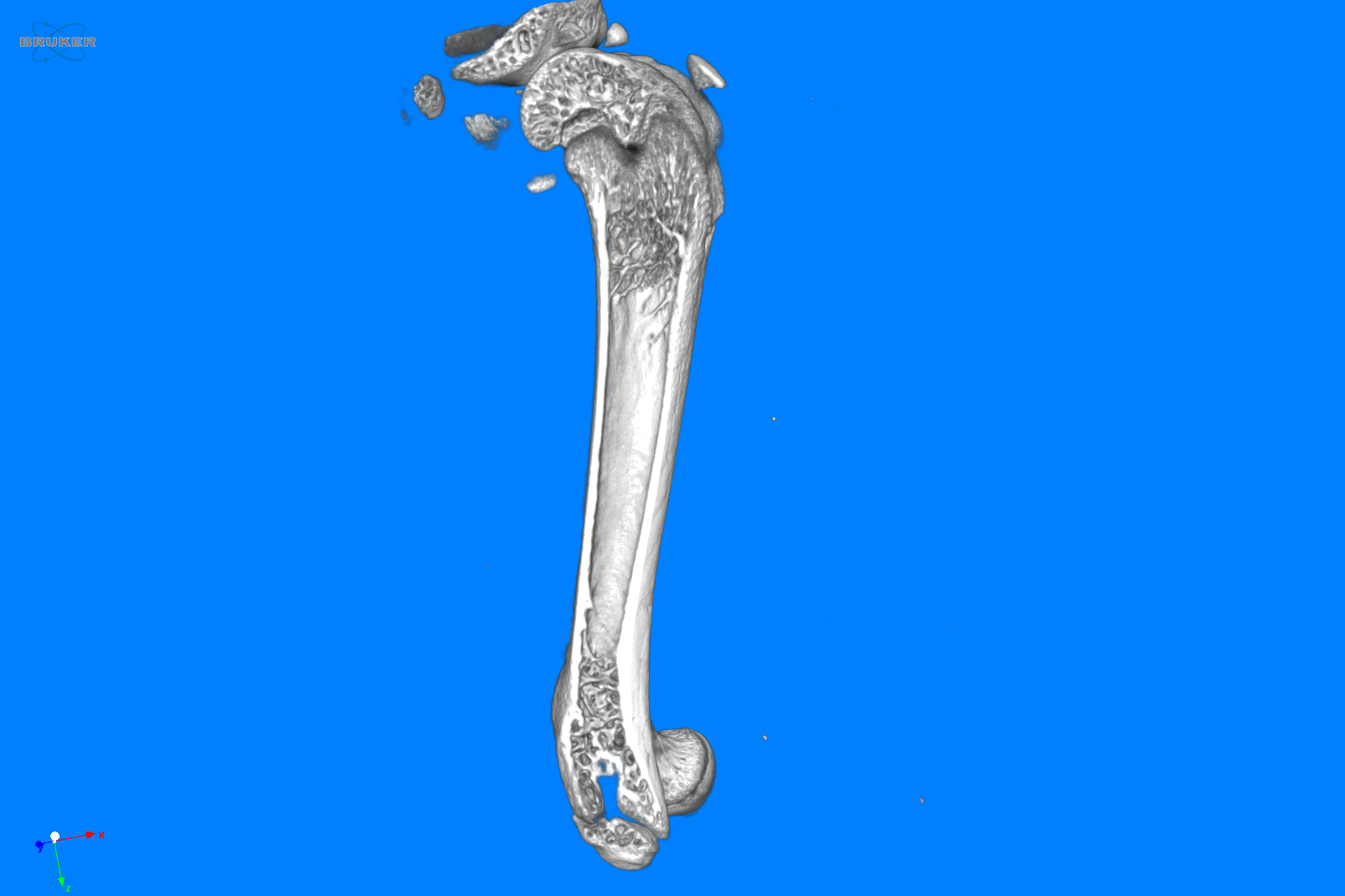

Supplement: Supplementary file 5 — Source data Fig. 2 [file 44318_2025_399_MOESM5_ESM.zip › Figure 2/2J/Fig2J-WT.tif]

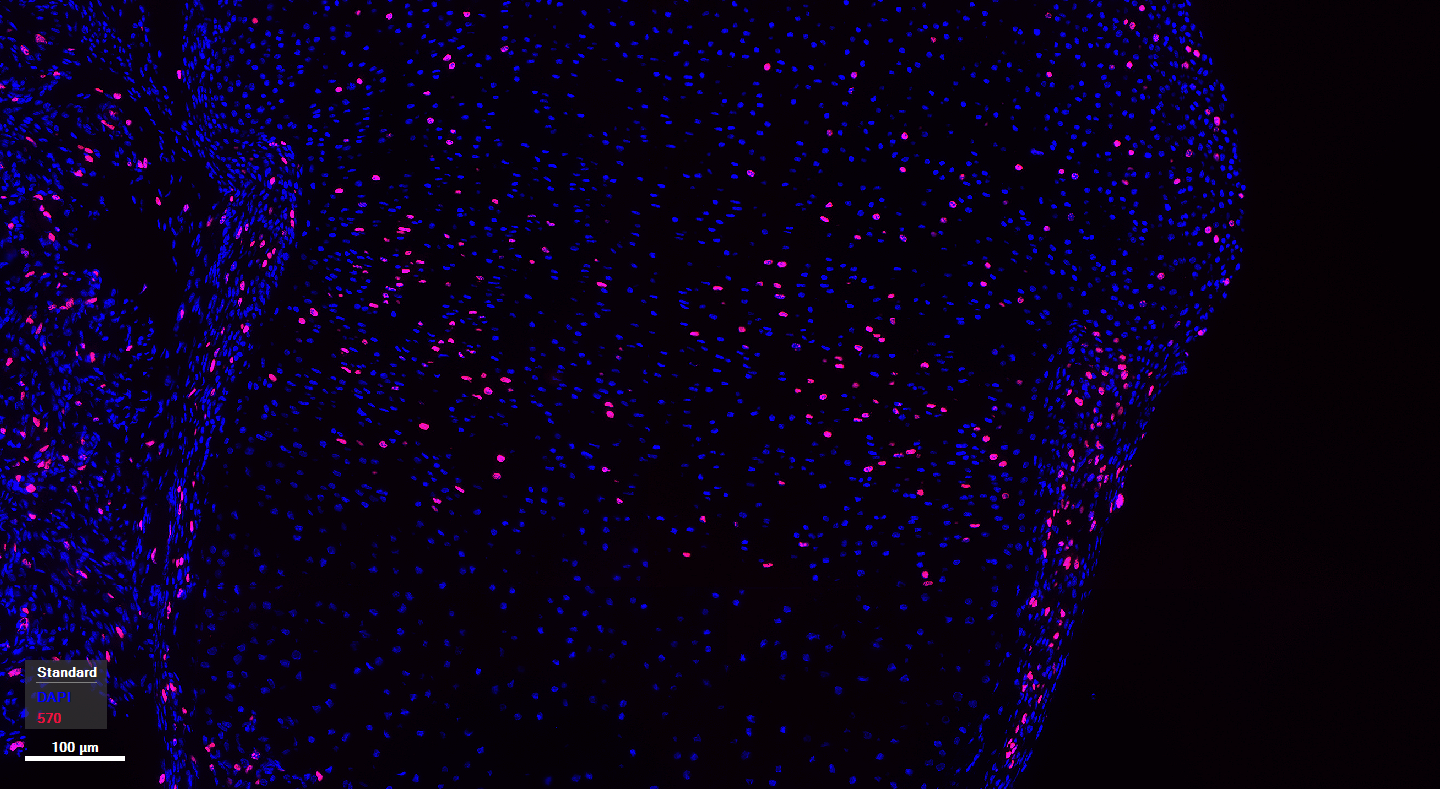

Supplement: Supplementary file 6 — Source data Fig. 3 [file 44318_2025_399_MOESM6_ESM.zip › Figure 3/3G/P3-Mettl3KO_10x.tif]

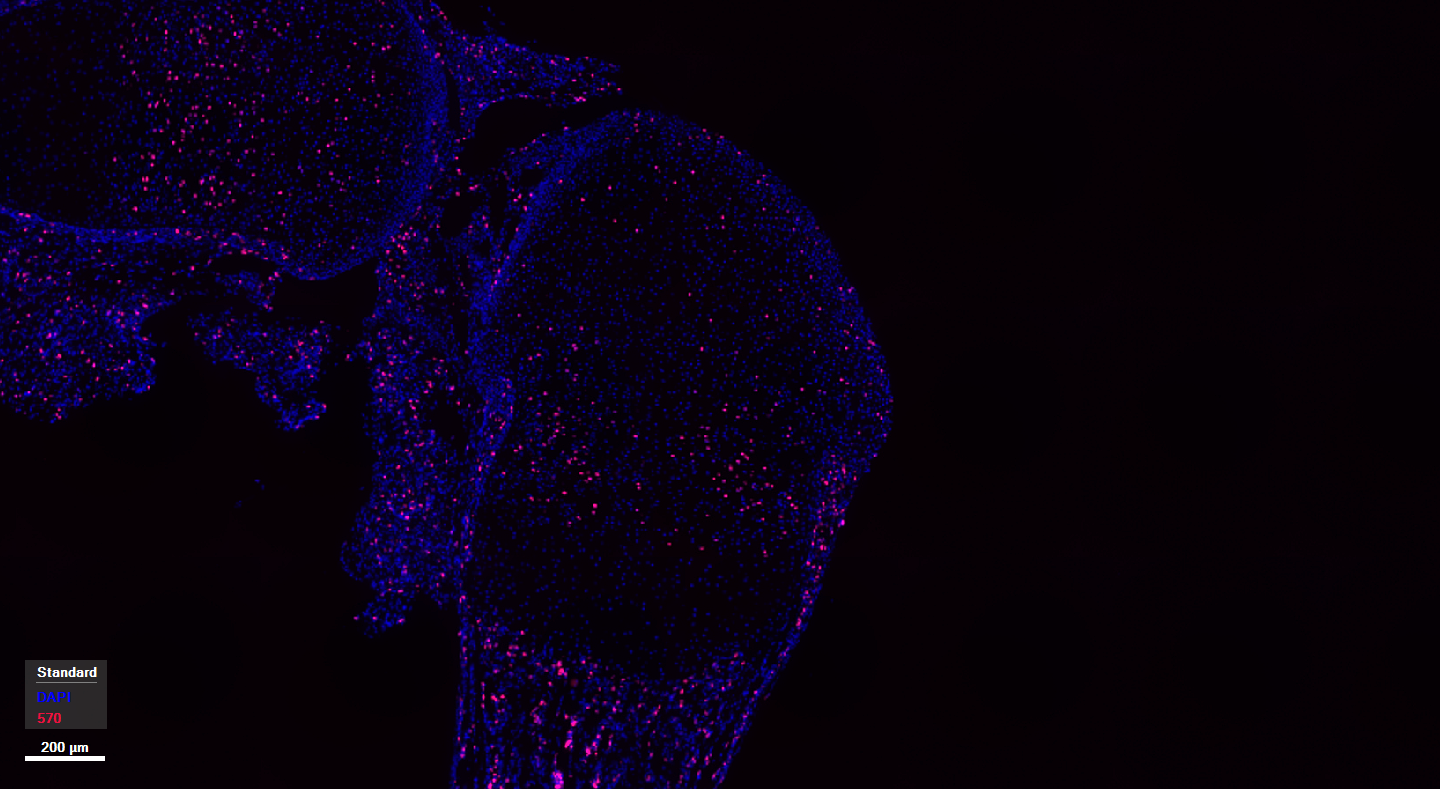

Supplement: Supplementary file 6 — Source data Fig. 3 [file 44318_2025_399_MOESM6_ESM.zip › Figure 3/3G/P3-Mettl3KO_4x.tif]

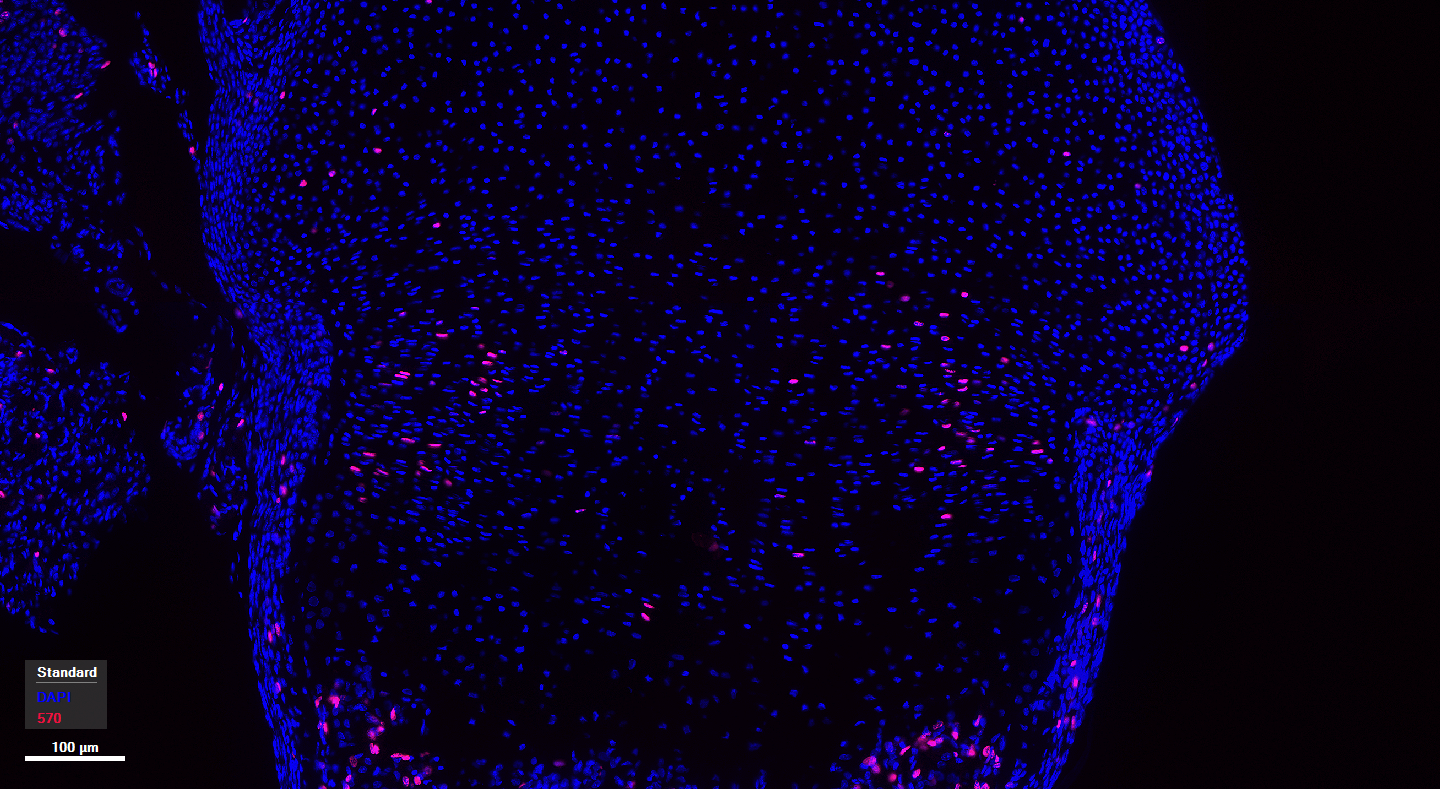

Supplement: Supplementary file 6 — Source data Fig. 3 [file 44318_2025_399_MOESM6_ESM.zip › Figure 3/3G/P3-WT_10x.tif]

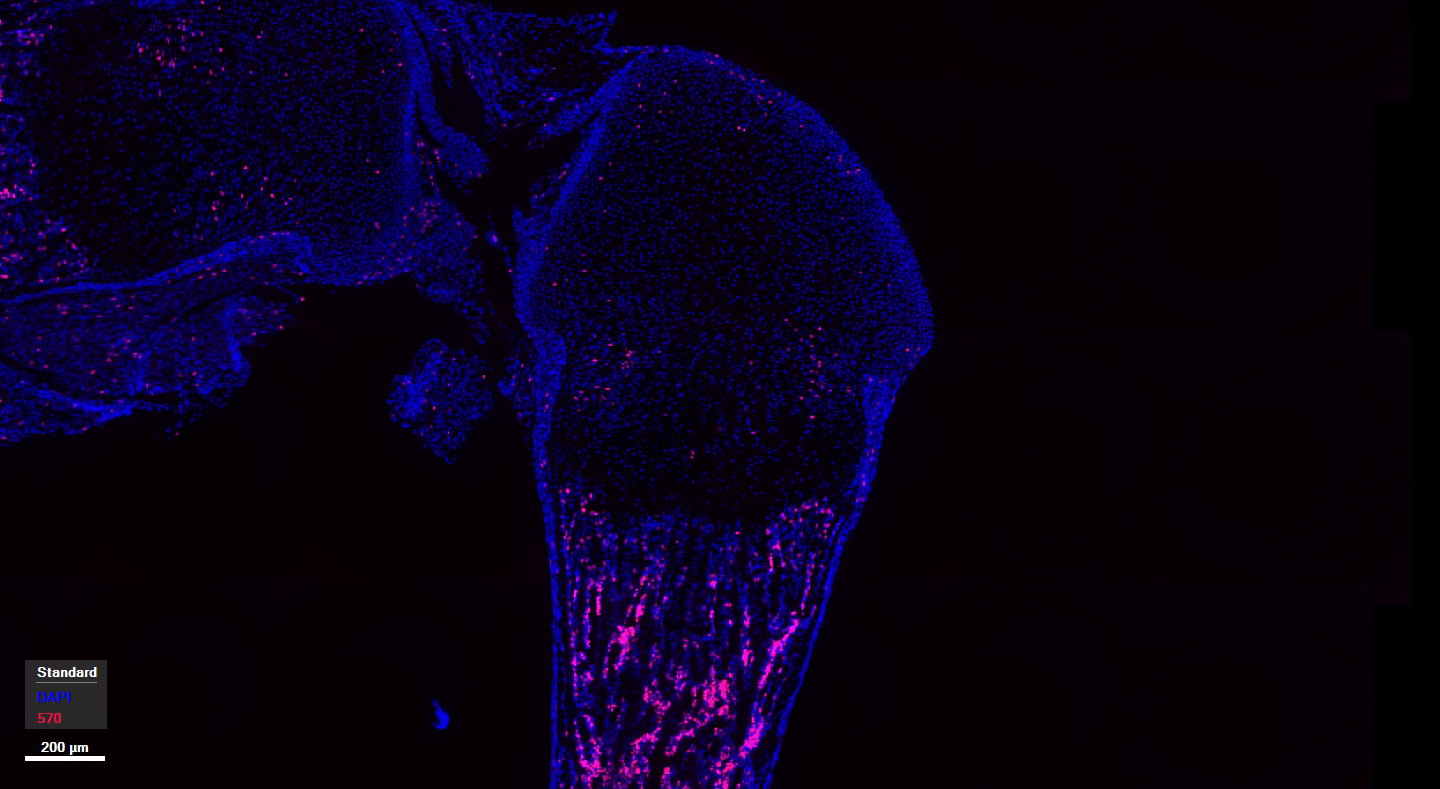

Supplement: Supplementary file 6 — Source data Fig. 3 [file 44318_2025_399_MOESM6_ESM.zip › Figure 3/3G/P3-WT_4x.tif]

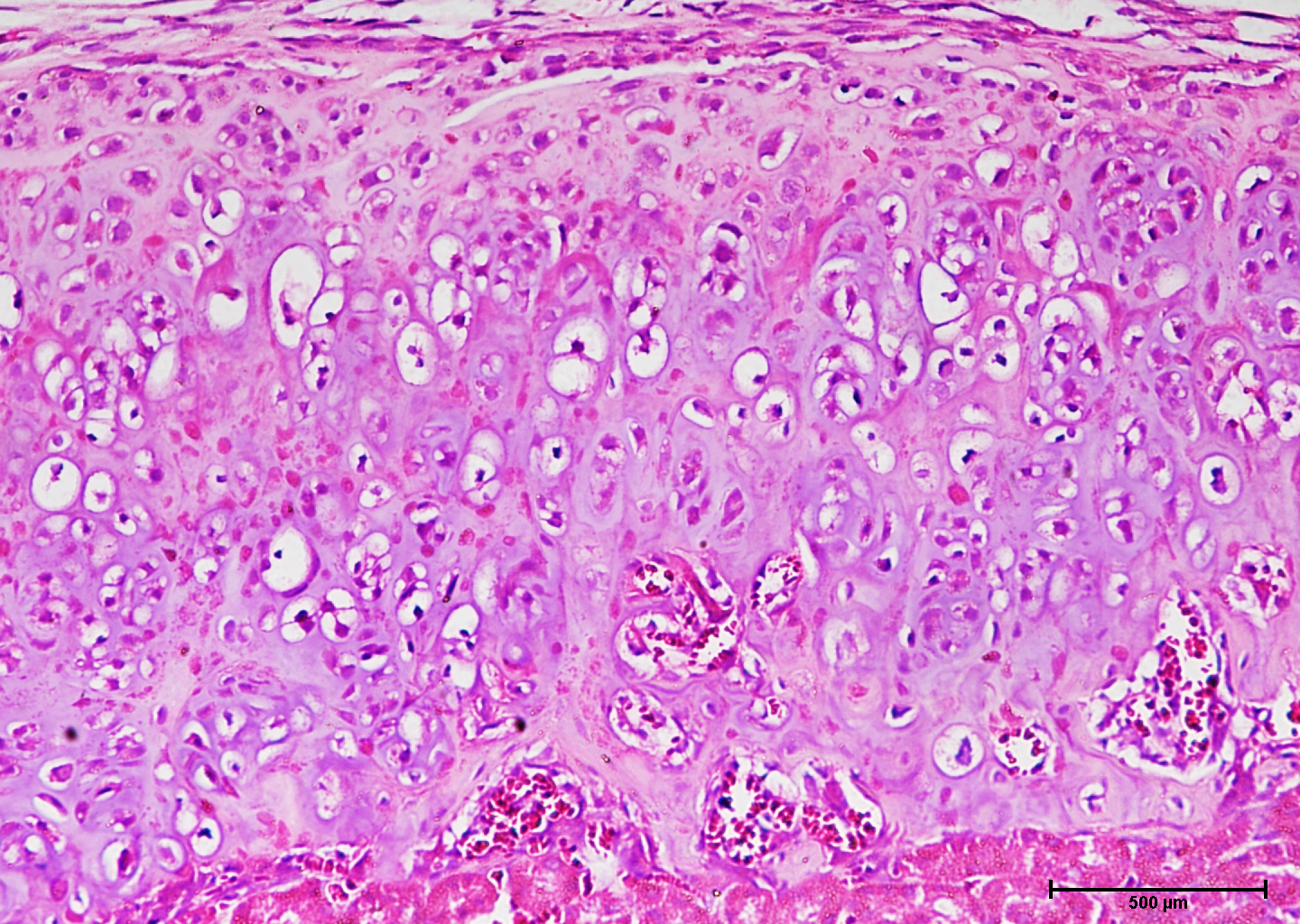

Supplement: Supplementary file 6 — Source data Fig. 3 [file 44318_2025_399_MOESM6_ESM.zip › Figure 3/3I/Figure3K-Mettl3KO.tif]

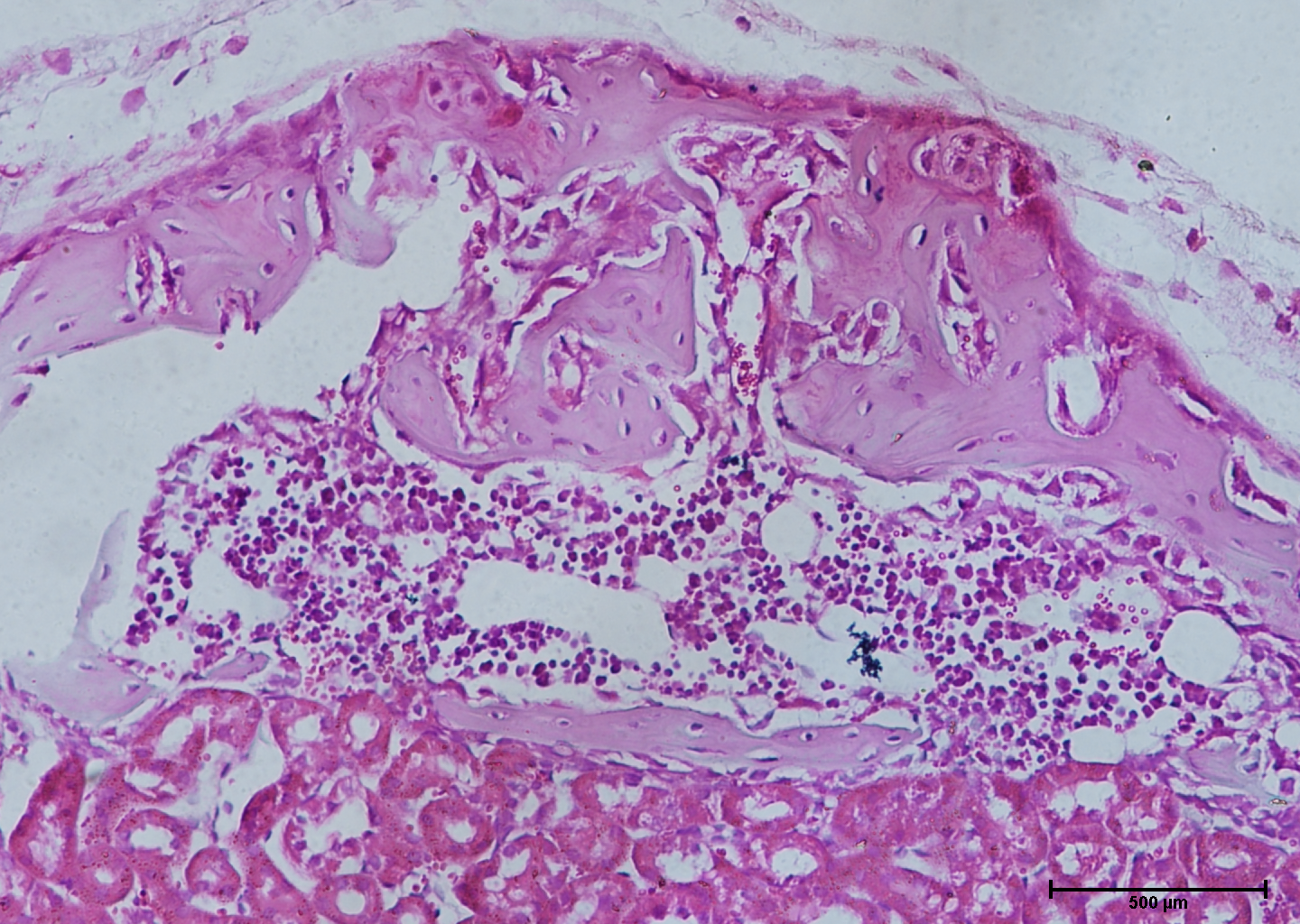

Supplement: Supplementary file 6 — Source data Fig. 3 [file 44318_2025_399_MOESM6_ESM.zip › Figure 3/3I/Figure3K-WT.tif]

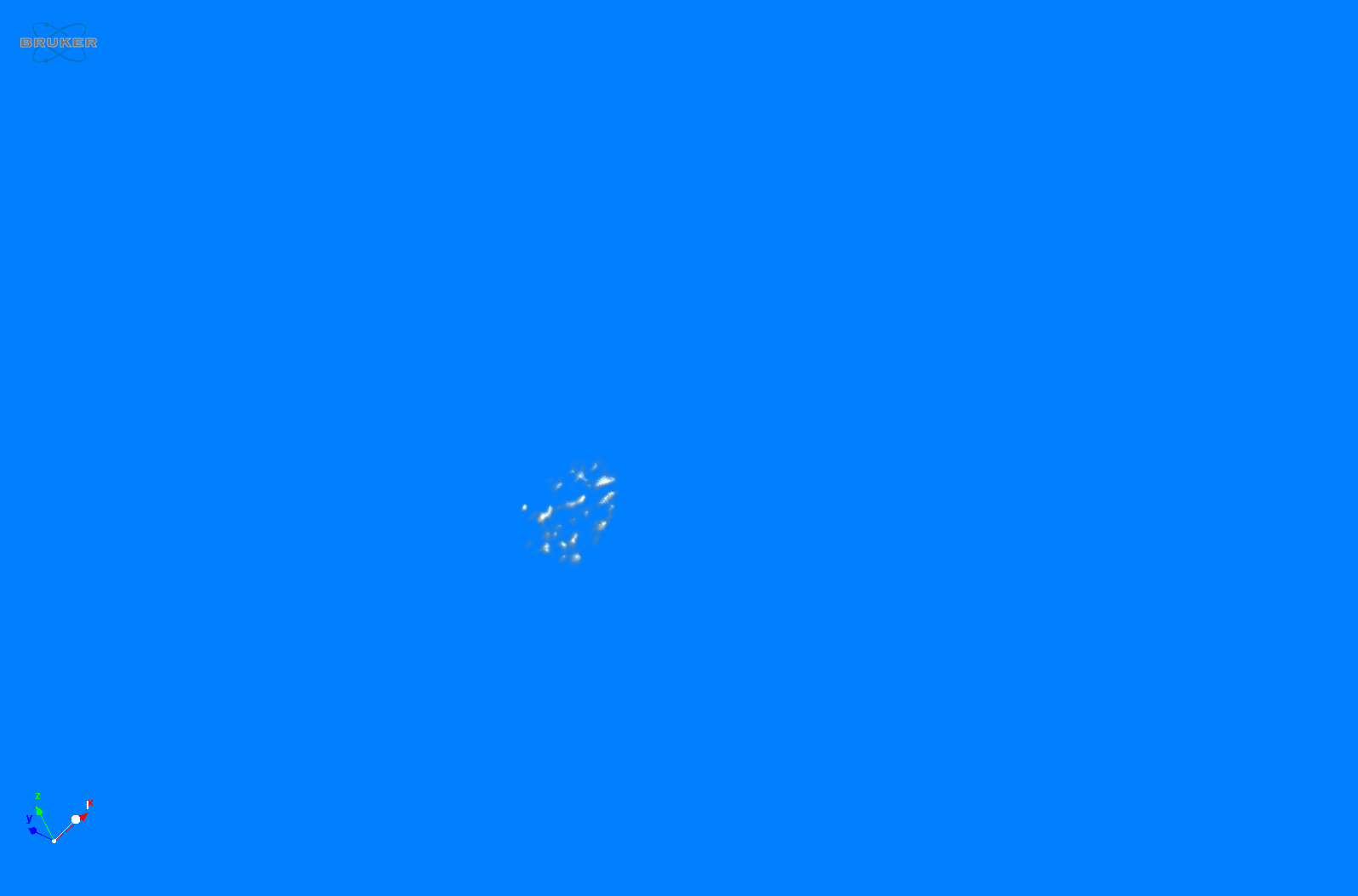

Supplement: Supplementary file 6 — Source data Fig. 3 [file 44318_2025_399_MOESM6_ESM.zip › Figure 3/3K/Figure 3K-Mettl3KO.tif]

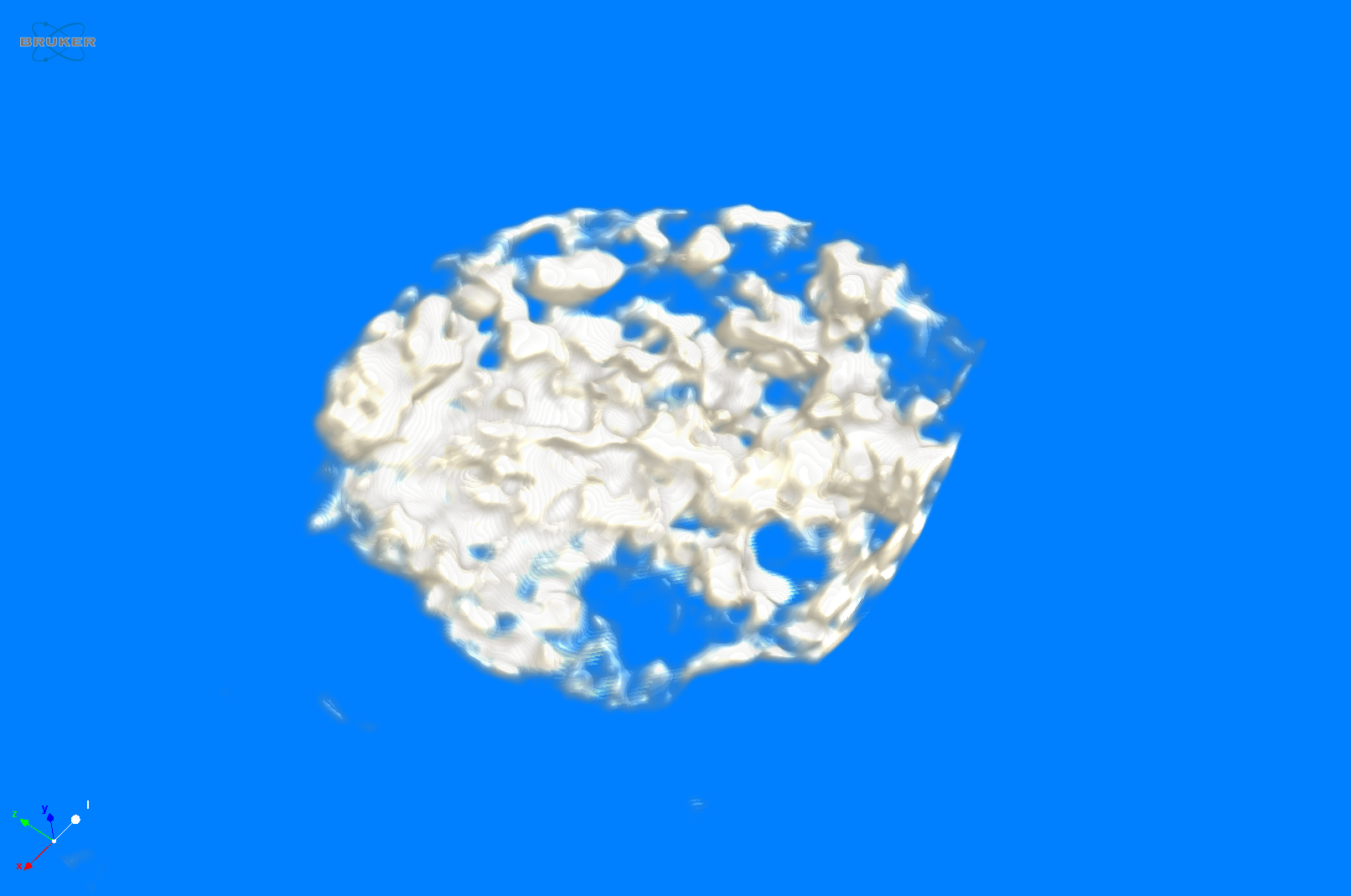

Supplement: Supplementary file 6 — Source data Fig. 3 [file 44318_2025_399_MOESM6_ESM.zip › Figure 3/3K/Figure 3K-WT.tif]

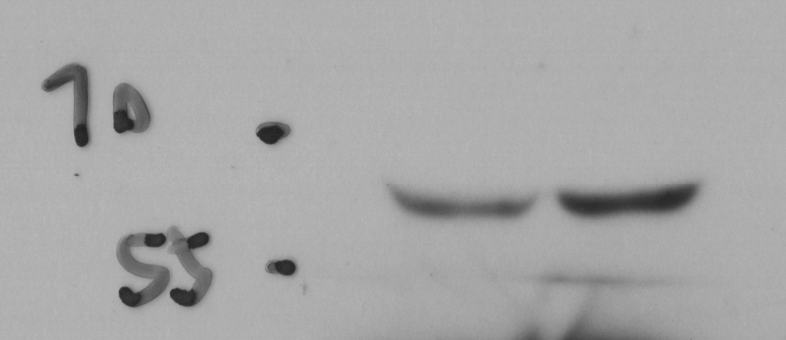

Supplement: Supplementary file 7 — Source data Fig. 4 [file 44318_2025_399_MOESM7_ESM.zip › Figure 4/4D/Figure 4D-Fem1b.tif]

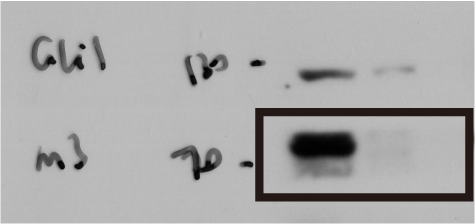

Supplement: Supplementary file 7 — Source data Fig. 4 [file 44318_2025_399_MOESM7_ESM.zip › Figure 4/4D/Figure 4D-Mettl3.tif]

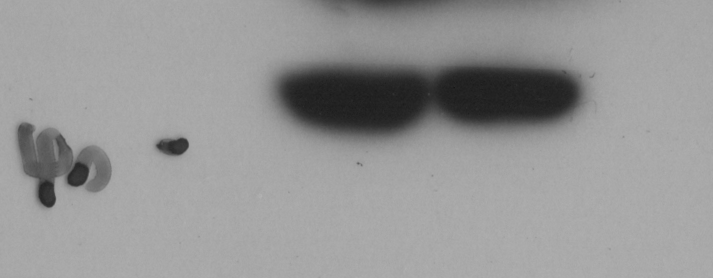

Supplement: Supplementary file 7 — Source data Fig. 4 [file 44318_2025_399_MOESM7_ESM.zip › Figure 4/4D/Figure 4D-Tubulin.tif]

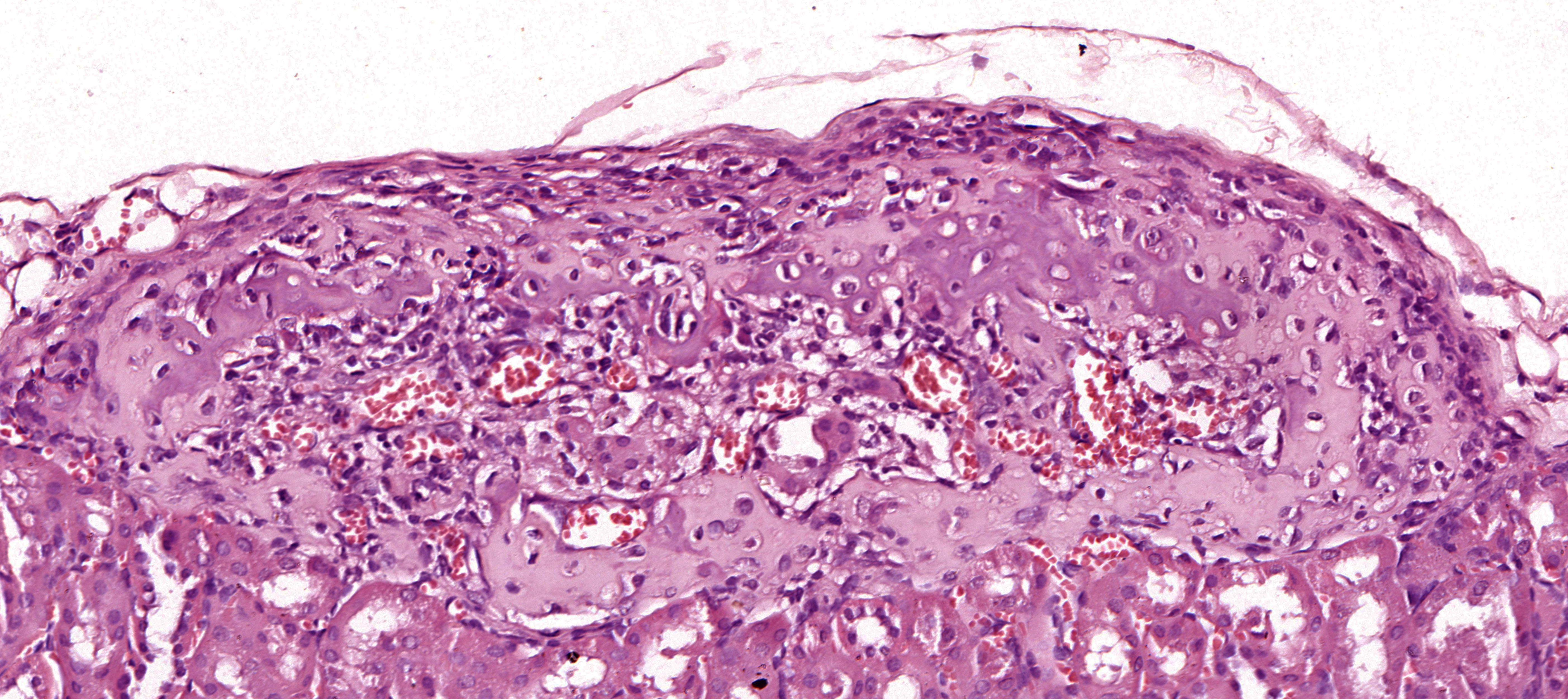

Supplement: Supplementary file 7 — Source data Fig. 4 [file 44318_2025_399_MOESM7_ESM.zip › Figure 4/4M/Figure 4M-Mettl3KO+shFem1b.tif]

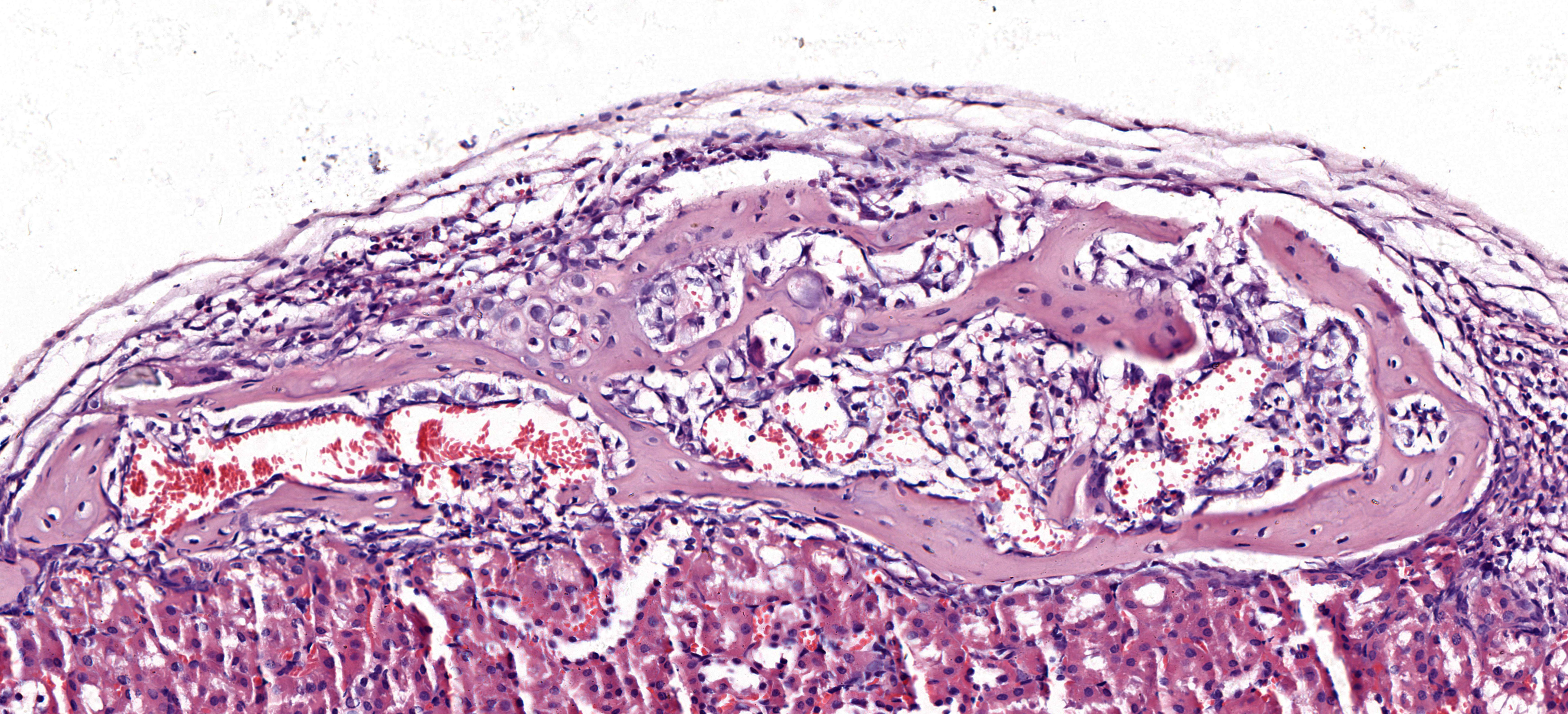

Supplement: Supplementary file 7 — Source data Fig. 4 [file 44318_2025_399_MOESM7_ESM.zip › Figure 4/4M/Figure 4M-WT+con.tif]

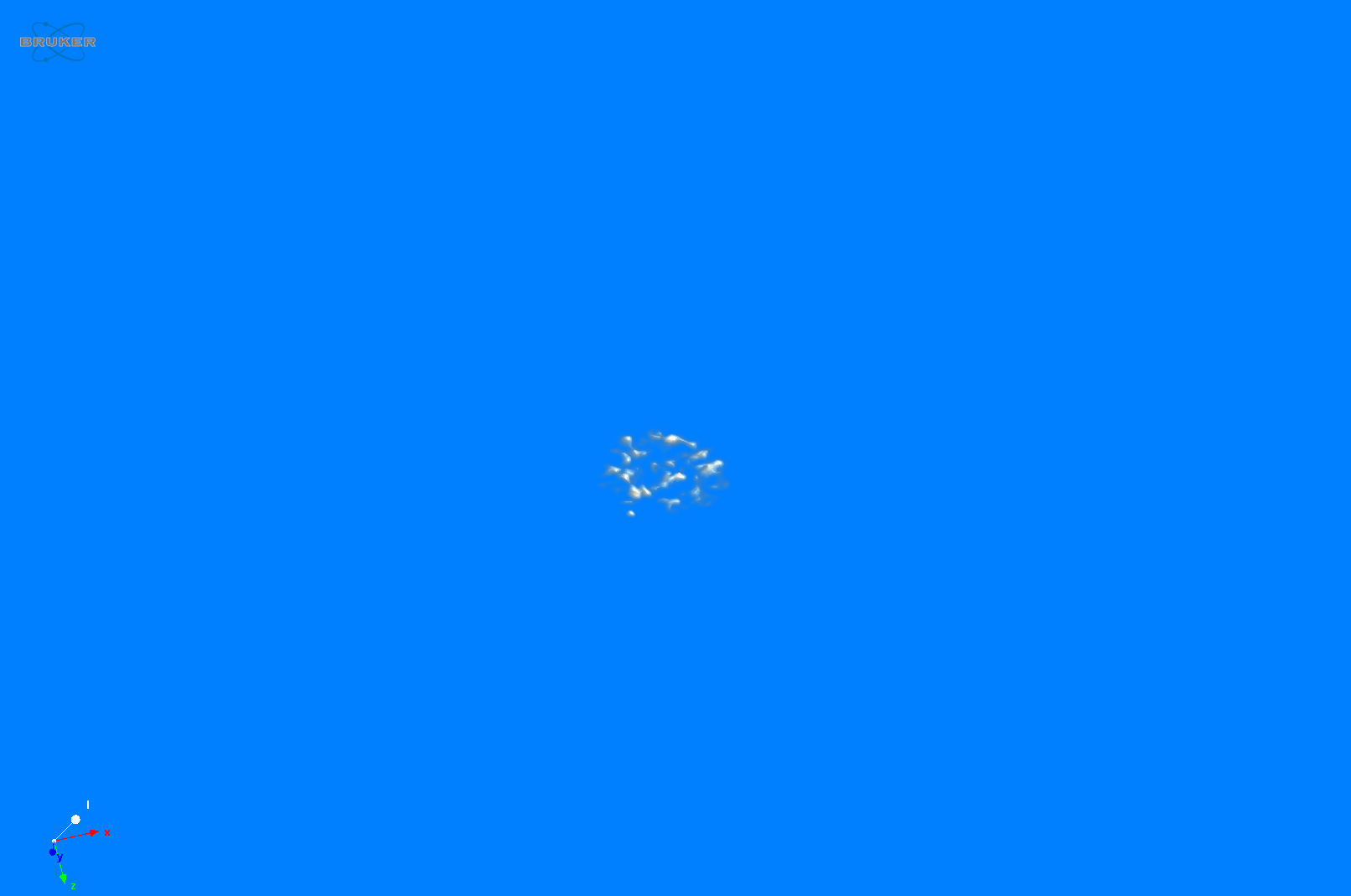

Supplement: Supplementary file 7 — Source data Fig. 4 [file 44318_2025_399_MOESM7_ESM.zip › Figure 4/4N/Figure 4N-ko+con.tif]

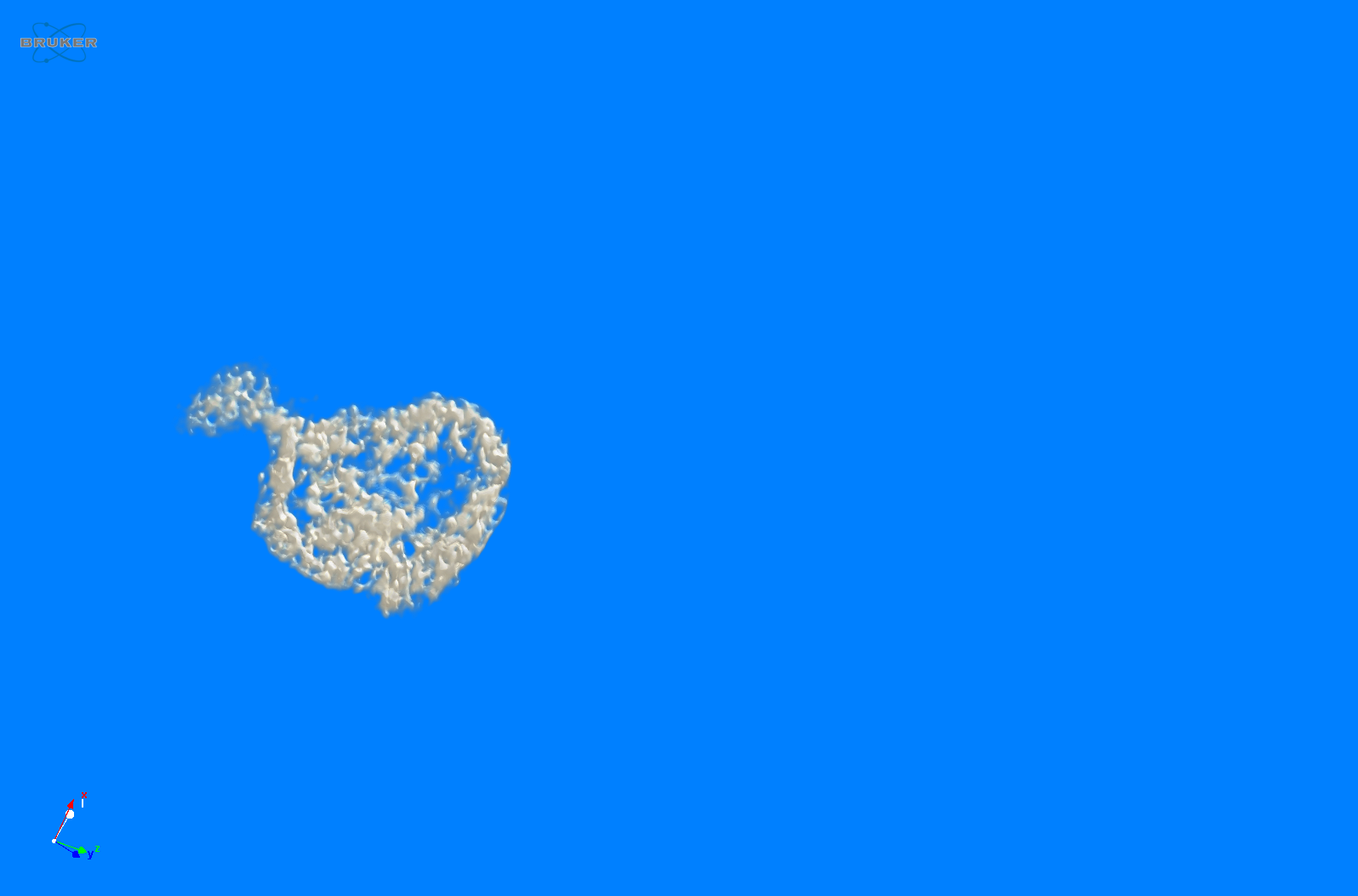

Supplement: Supplementary file 7 — Source data Fig. 4 [file 44318_2025_399_MOESM7_ESM.zip › Figure 4/4N/Figure 4N-ko+shFem1b.tif]

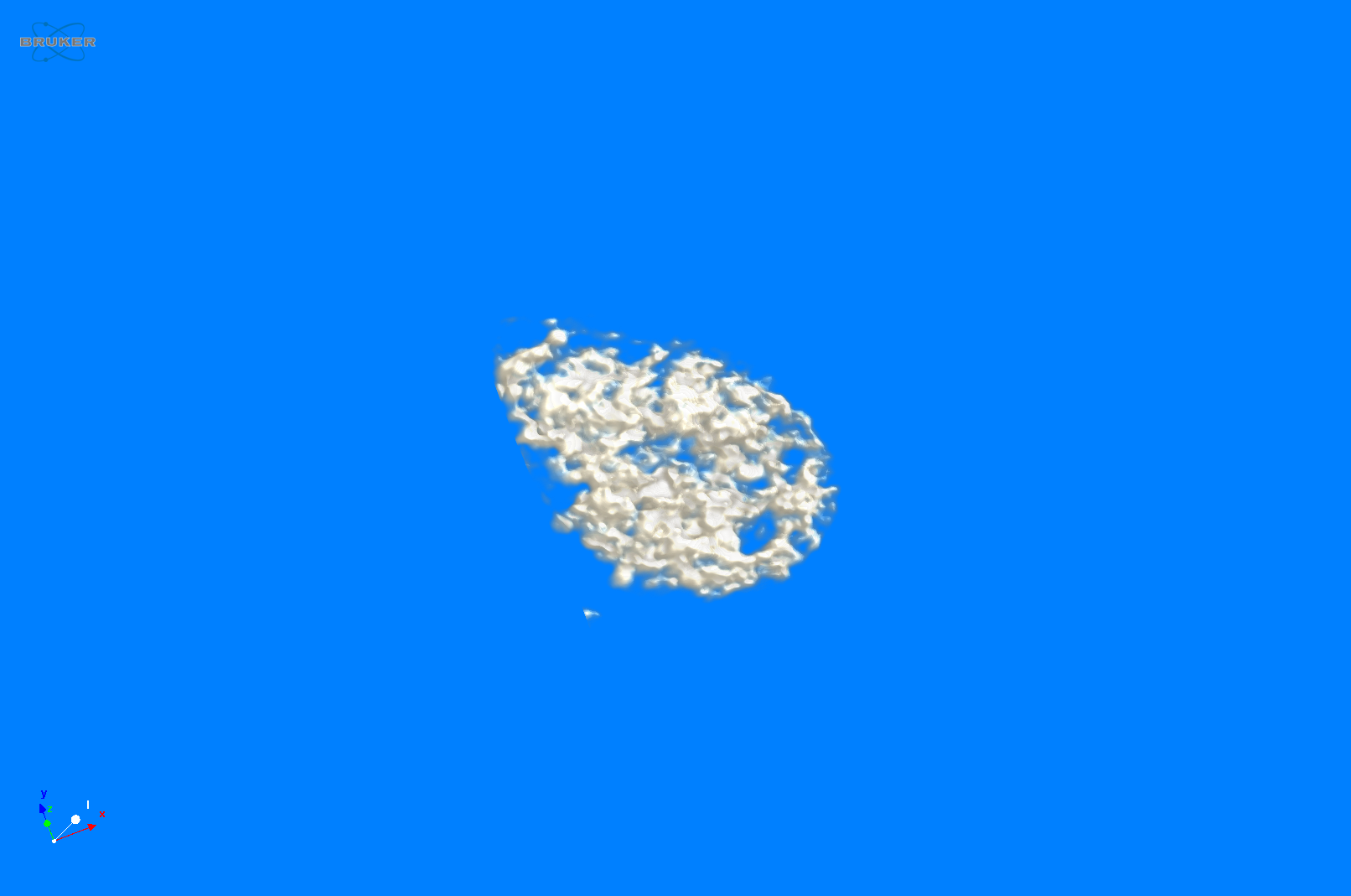

Supplement: Supplementary file 7 — Source data Fig. 4 [file 44318_2025_399_MOESM7_ESM.zip › Figure 4/4N/Figure 4N-wt+con.tif]

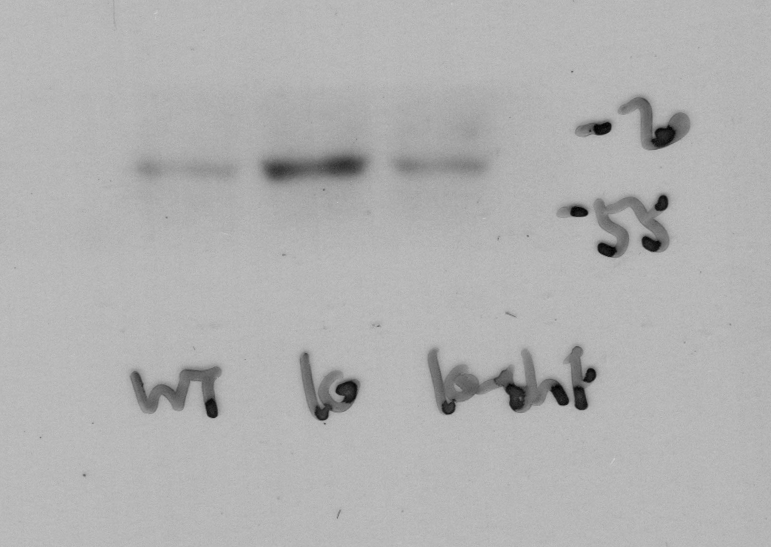

Supplement: Supplementary file 8 — Source data Fig. 5 [file 44318_2025_399_MOESM8_ESM.zip › Figure 5/5A/Figure 5A-Fem1b1.tif]

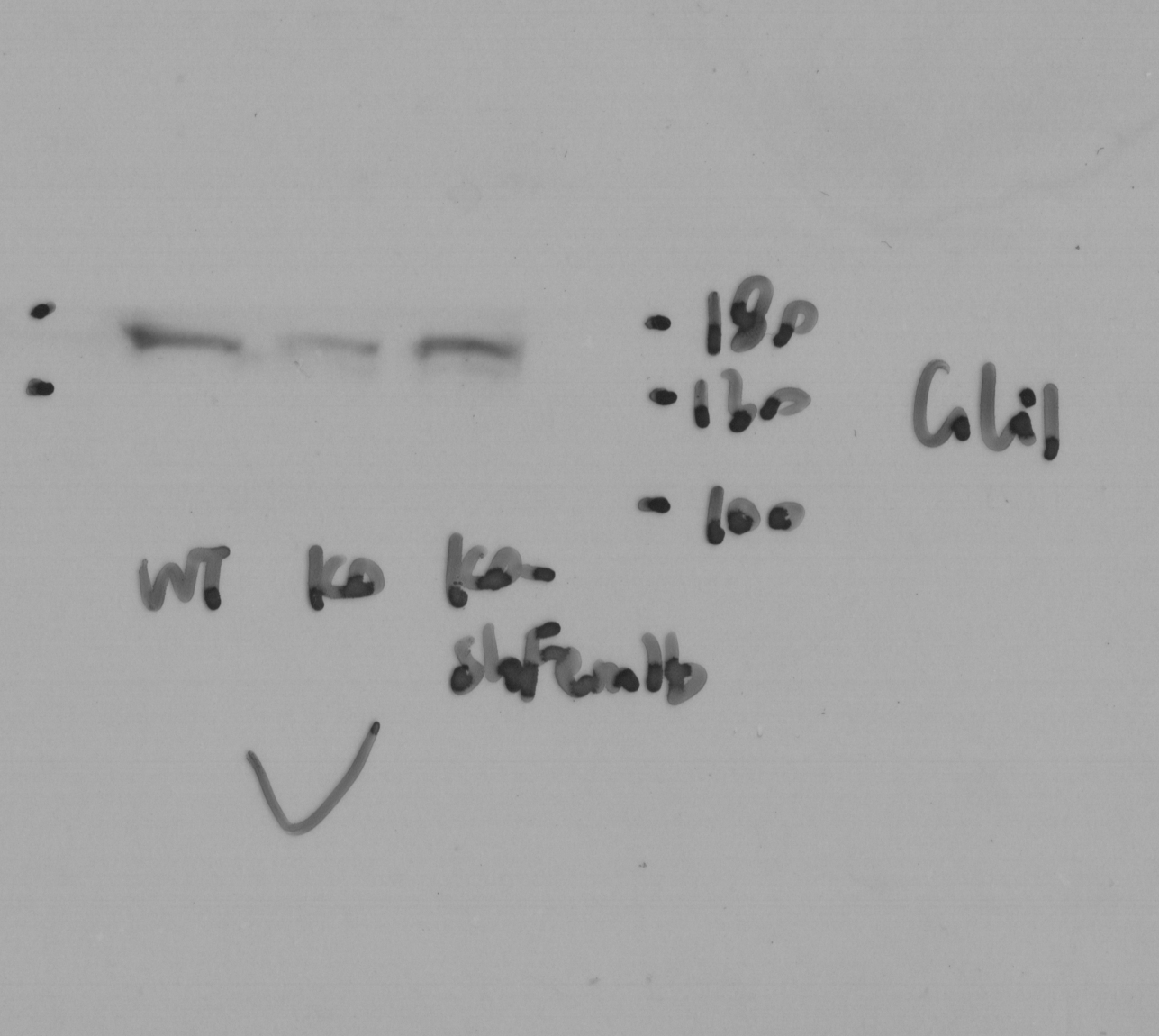

Supplement: Supplementary file 8 — Source data Fig. 5 [file 44318_2025_399_MOESM8_ESM.zip › Figure 5/5A/Figure 5A-Gli1.tif]

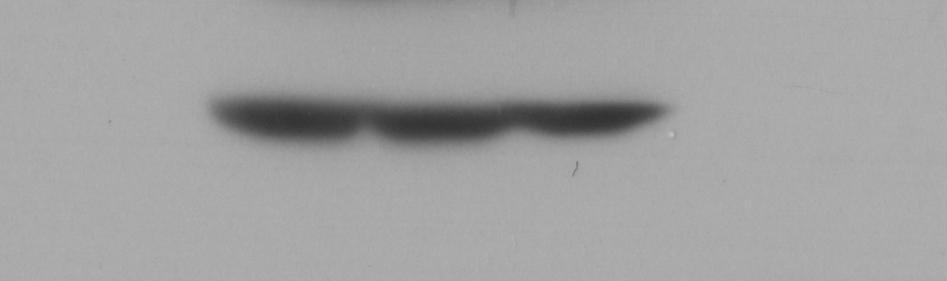

Supplement: Supplementary file 8 — Source data Fig. 5 [file 44318_2025_399_MOESM8_ESM.zip › Figure 5/5A/Figure 5A-Tubulin.tif]

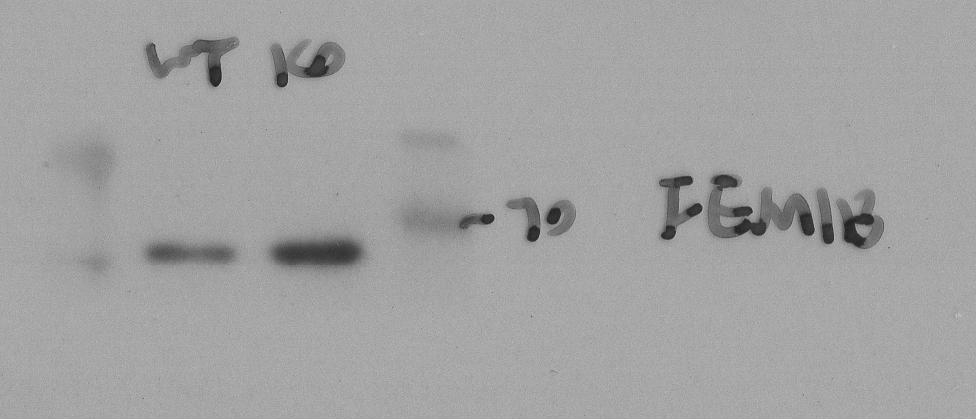

Supplement: Supplementary file 8 — Source data Fig. 5 [file 44318_2025_399_MOESM8_ESM.zip › Figure 5/5B/Figure 5B-Fem1b-input.tif]

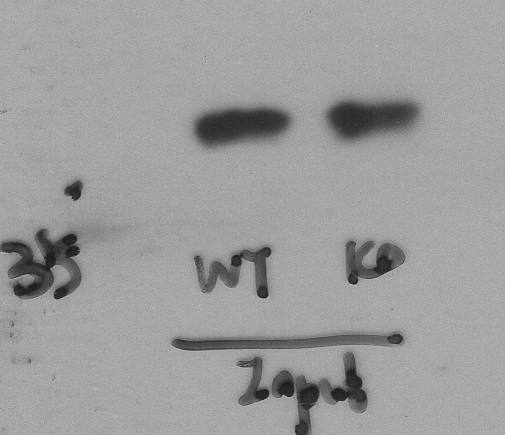

Supplement: Supplementary file 8 — Source data Fig. 5 [file 44318_2025_399_MOESM8_ESM.zip › Figure 5/5B/Figure 5B-Gapdh-input.tif]

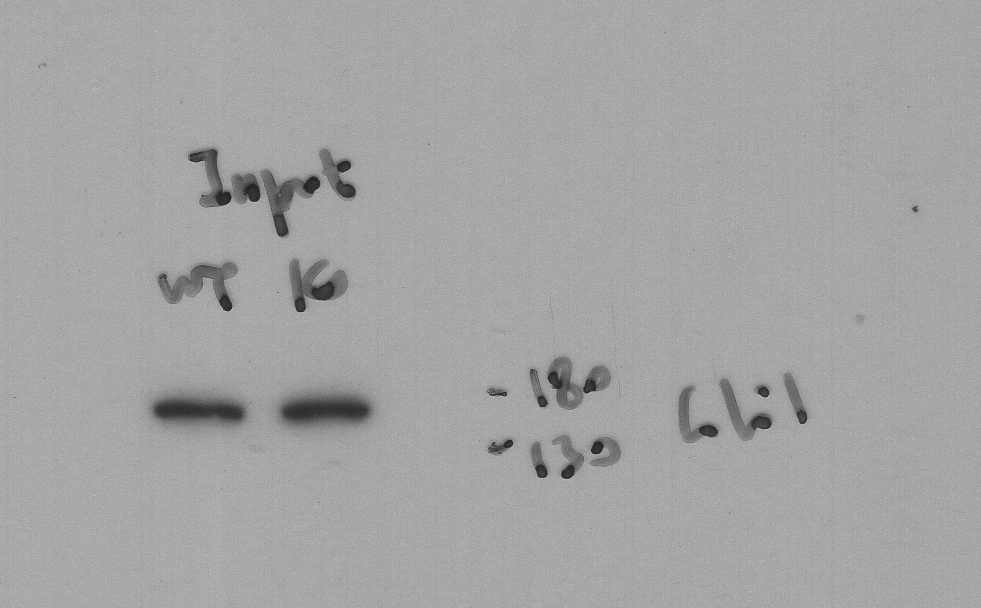

Supplement: Supplementary file 8 — Source data Fig. 5 [file 44318_2025_399_MOESM8_ESM.zip › Figure 5/5B/Figure 5B-Gli1-input.tif]

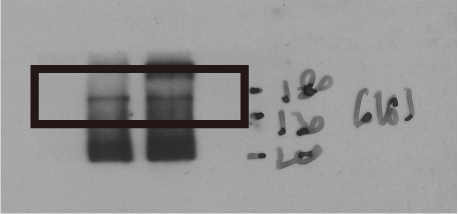

Supplement: Supplementary file 8 — Source data Fig. 5 [file 44318_2025_399_MOESM8_ESM.zip › Figure 5/5B/Figure 5B-Gli1-ip.tif]

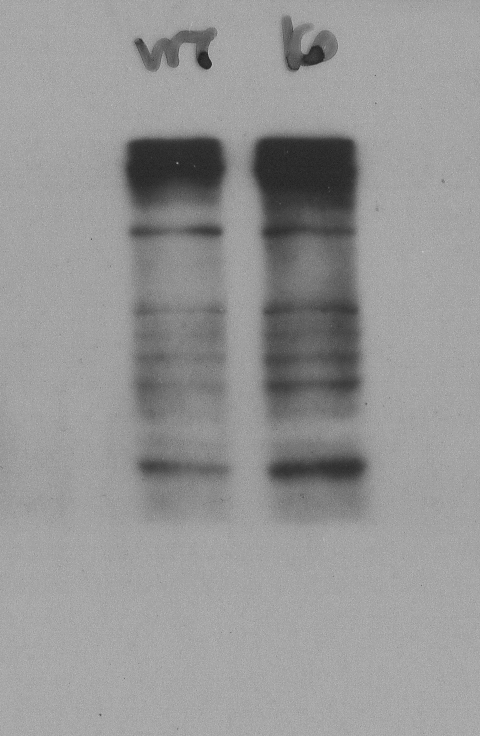

Supplement: Supplementary file 8 — Source data Fig. 5 [file 44318_2025_399_MOESM8_ESM.zip › Figure 5/5B/Figure 5B-Ub-input.tif]

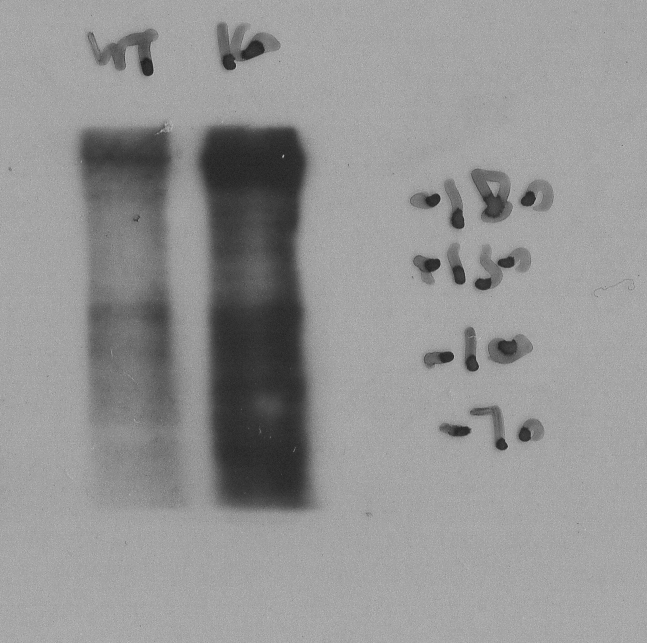

Supplement: Supplementary file 8 — Source data Fig. 5 [file 44318_2025_399_MOESM8_ESM.zip › Figure 5/5B/Figure 5B-Ub-ip.tif]

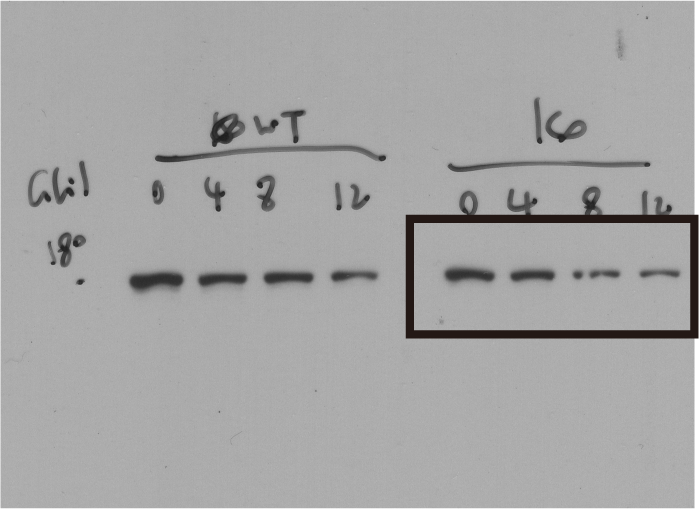

Supplement: Supplementary file 8 — Source data Fig. 5 [file 44318_2025_399_MOESM8_ESM.zip › Figure 5/5C/Figure 5C-Gli1-Mettl3KO.tif]

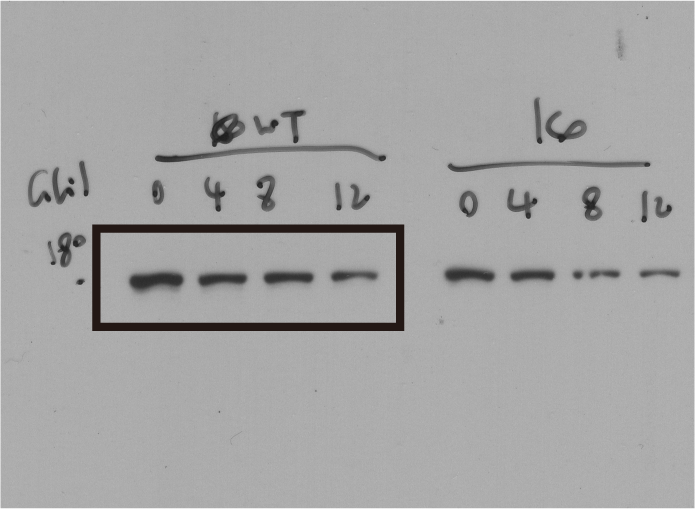

Supplement: Supplementary file 8 — Source data Fig. 5 [file 44318_2025_399_MOESM8_ESM.zip › Figure 5/5C/Figure 5C-Gli1-WT.tif]

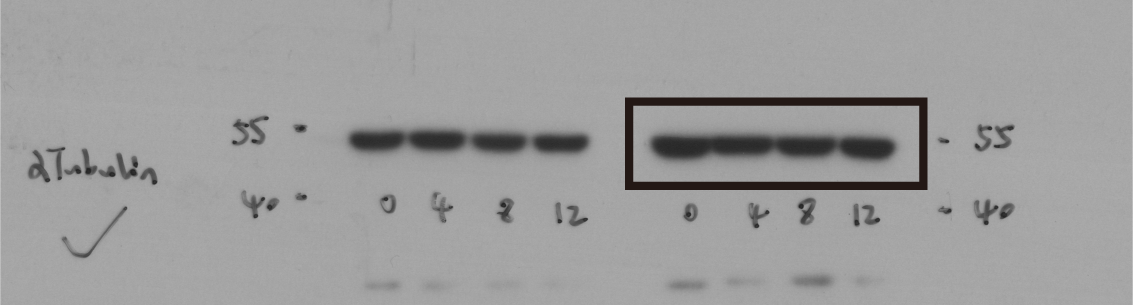

Supplement: Supplementary file 8 — Source data Fig. 5 [file 44318_2025_399_MOESM8_ESM.zip › Figure 5/5C/Figure 5C-Tubulin-Mettl3KO.tif]

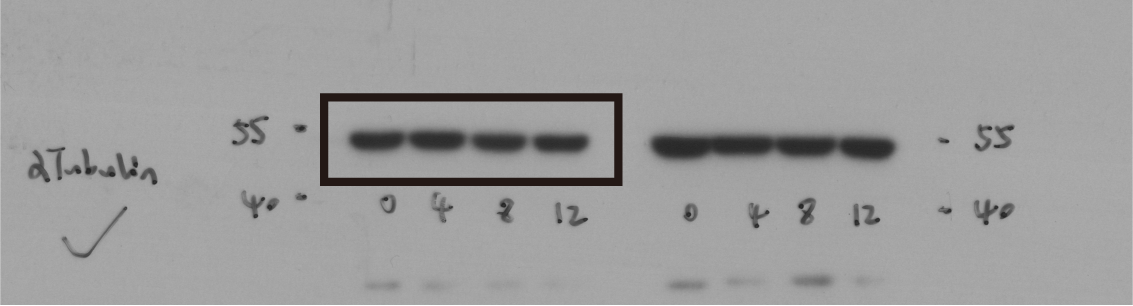

Supplement: Supplementary file 8 — Source data Fig. 5 [file 44318_2025_399_MOESM8_ESM.zip › Figure 5/5C/Figure 5C-Tubulin-WT.tif]

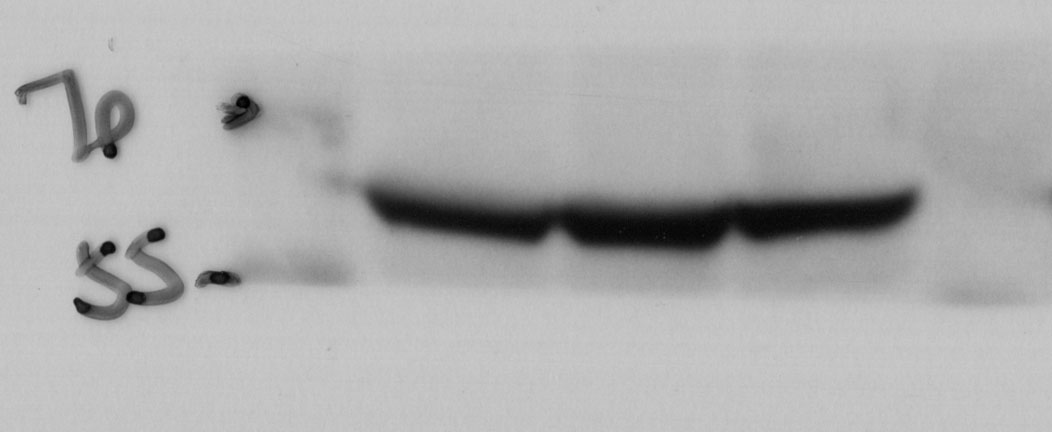

Supplement: Supplementary file 8 — Source data Fig. 5 [file 44318_2025_399_MOESM8_ESM.zip › Figure 5/5D/Figure 5D-Fem1b.tif]

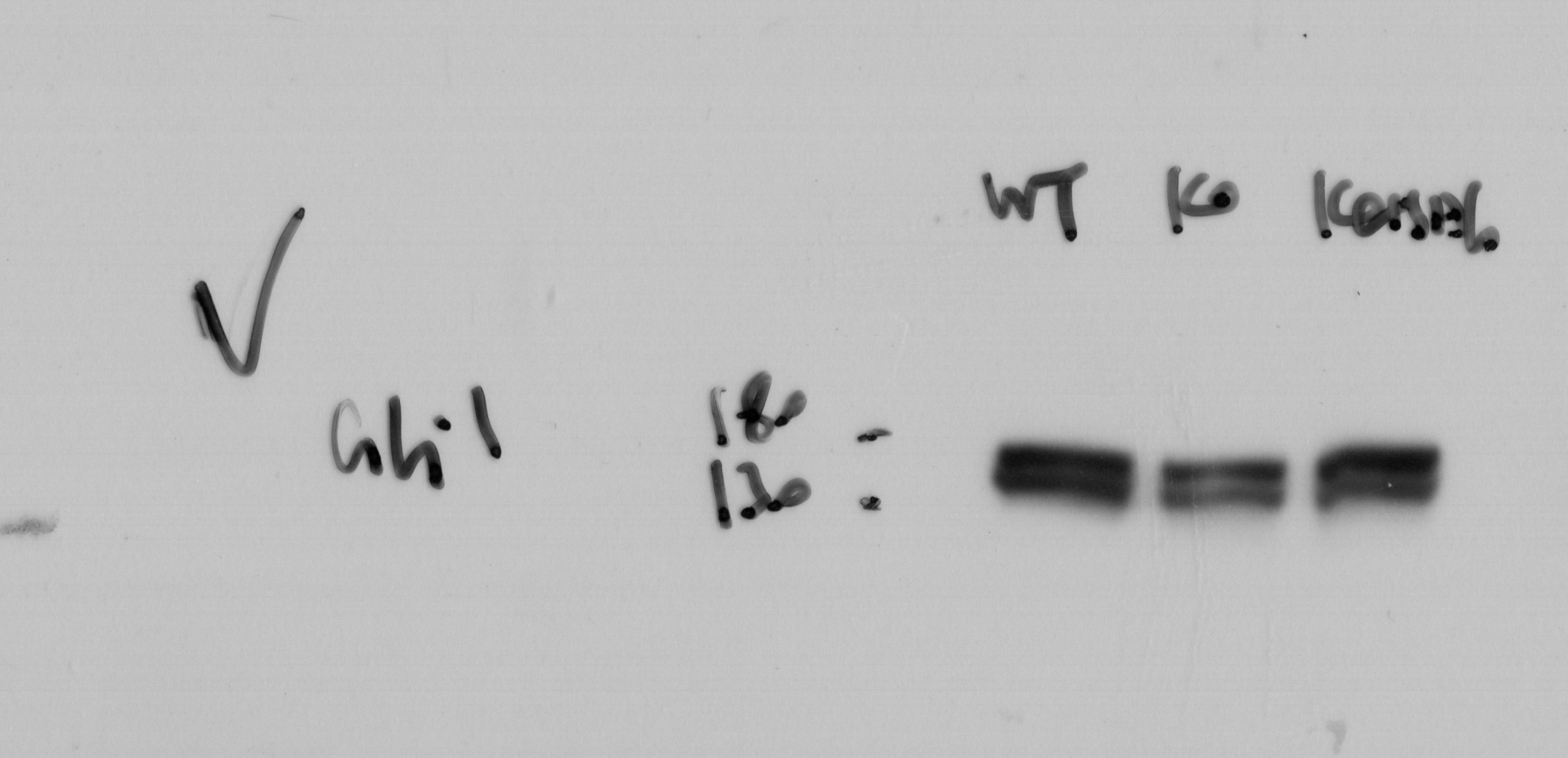

Supplement: Supplementary file 8 — Source data Fig. 5 [file 44318_2025_399_MOESM8_ESM.zip › Figure 5/5D/Figure 5D-Gli1.tif]

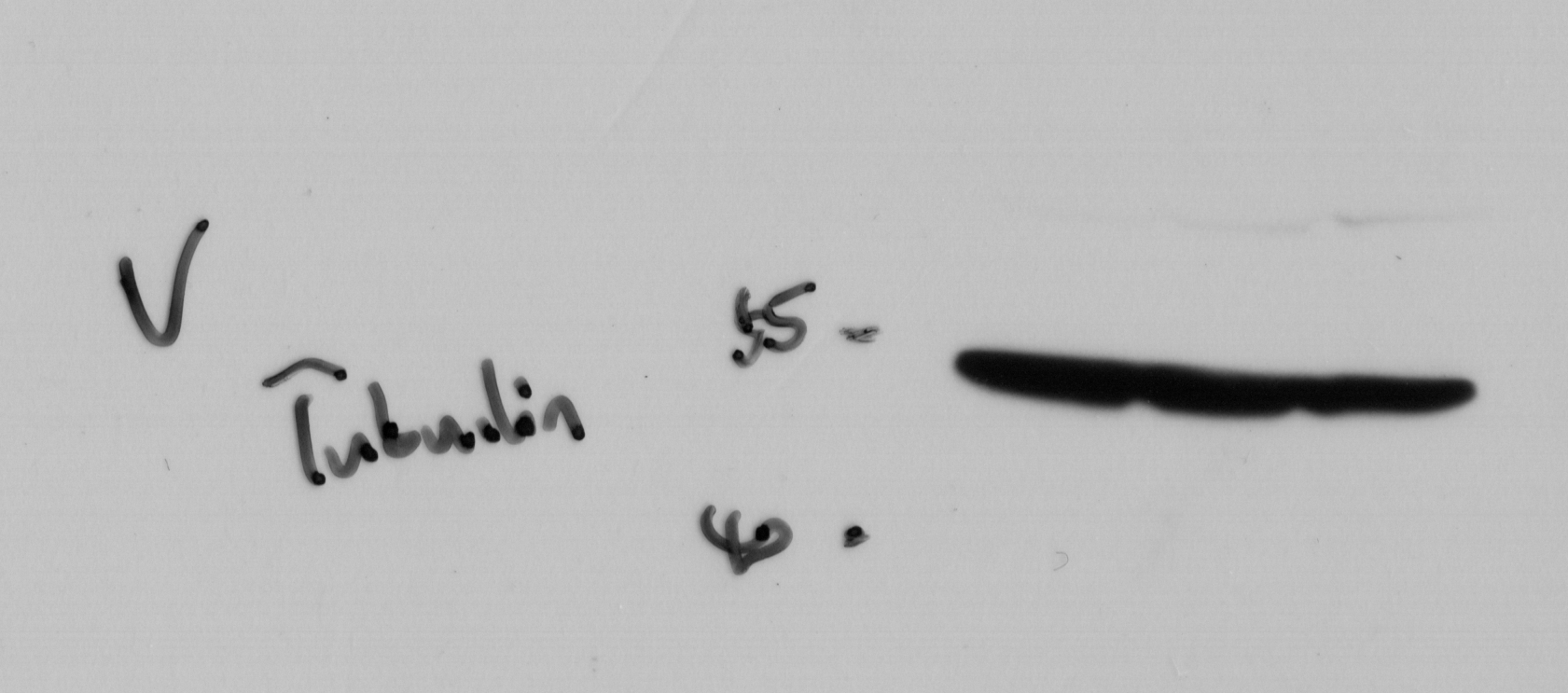

Supplement: Supplementary file 8 — Source data Fig. 5 [file 44318_2025_399_MOESM8_ESM.zip › Figure 5/5D/Figure 5D-Tubulin.tif]

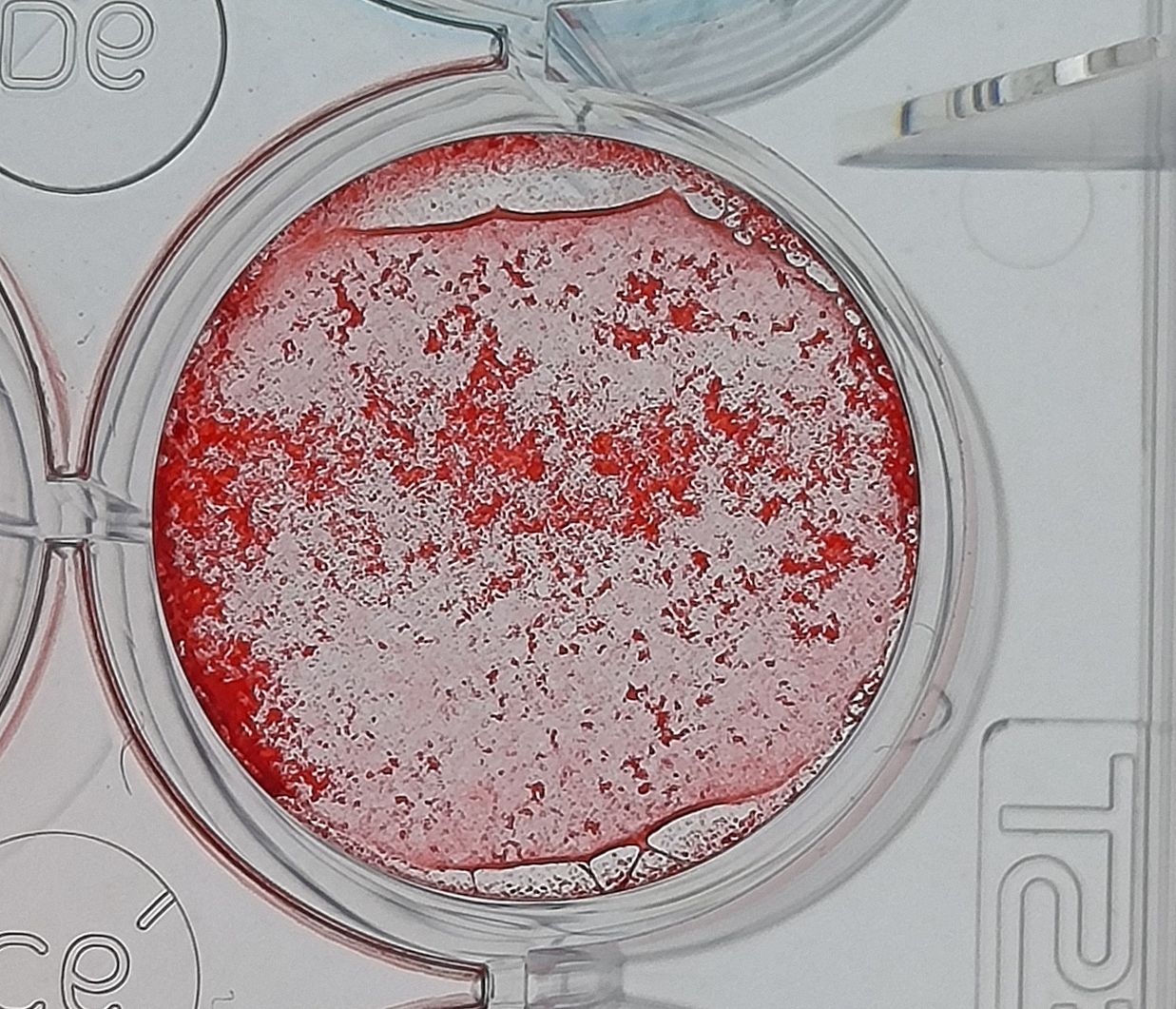

Supplement: Supplementary file 8 — Source data Fig. 5 [file 44318_2025_399_MOESM8_ESM.zip › Figure 5/5E/Figure5E-Mettl3KO+SAG-whole.tif]

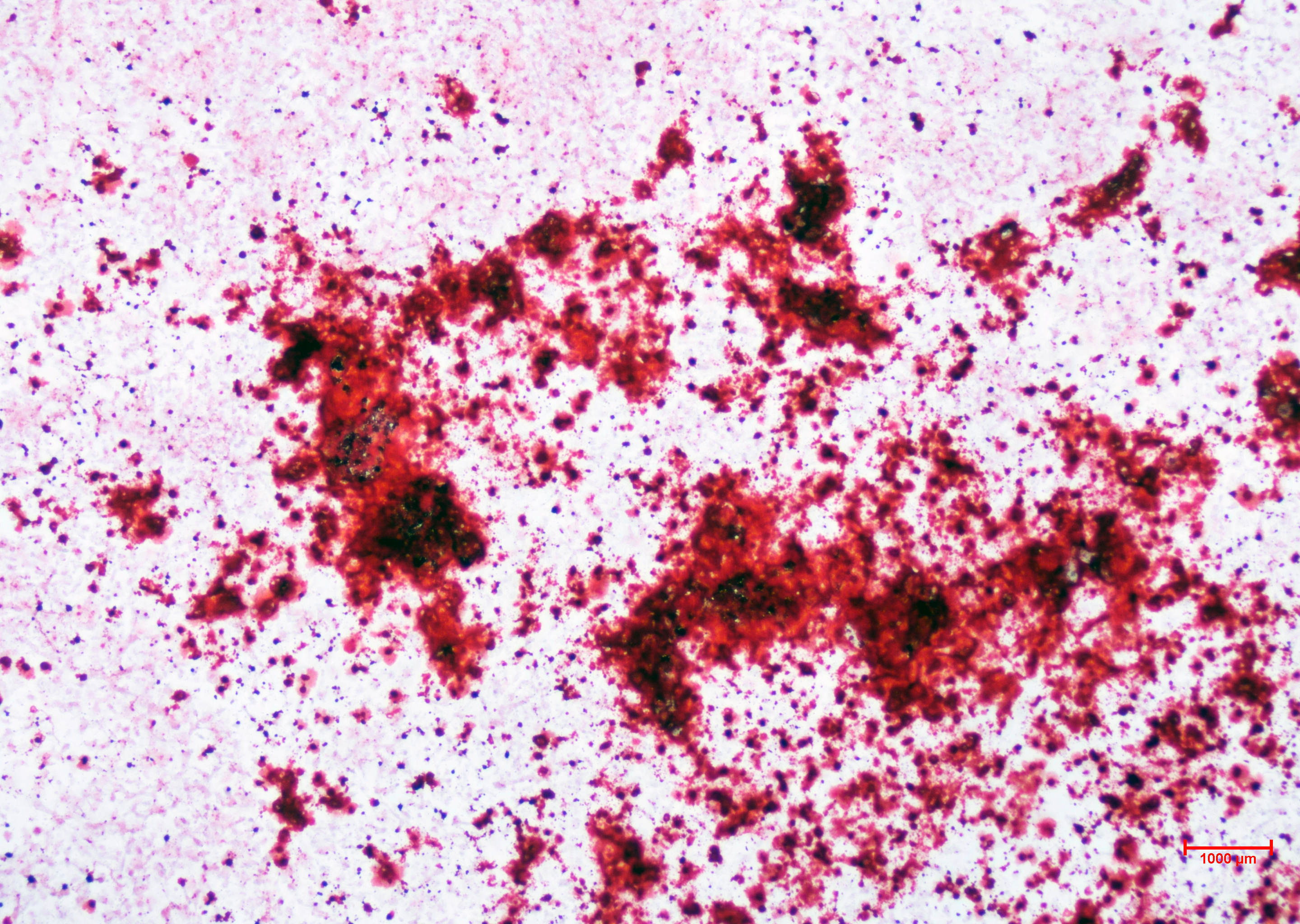

Supplement: Supplementary file 8 — Source data Fig. 5 [file 44318_2025_399_MOESM8_ESM.zip › Figure 5/5E/Figure5E-Mettl3KO+SAG.tif]

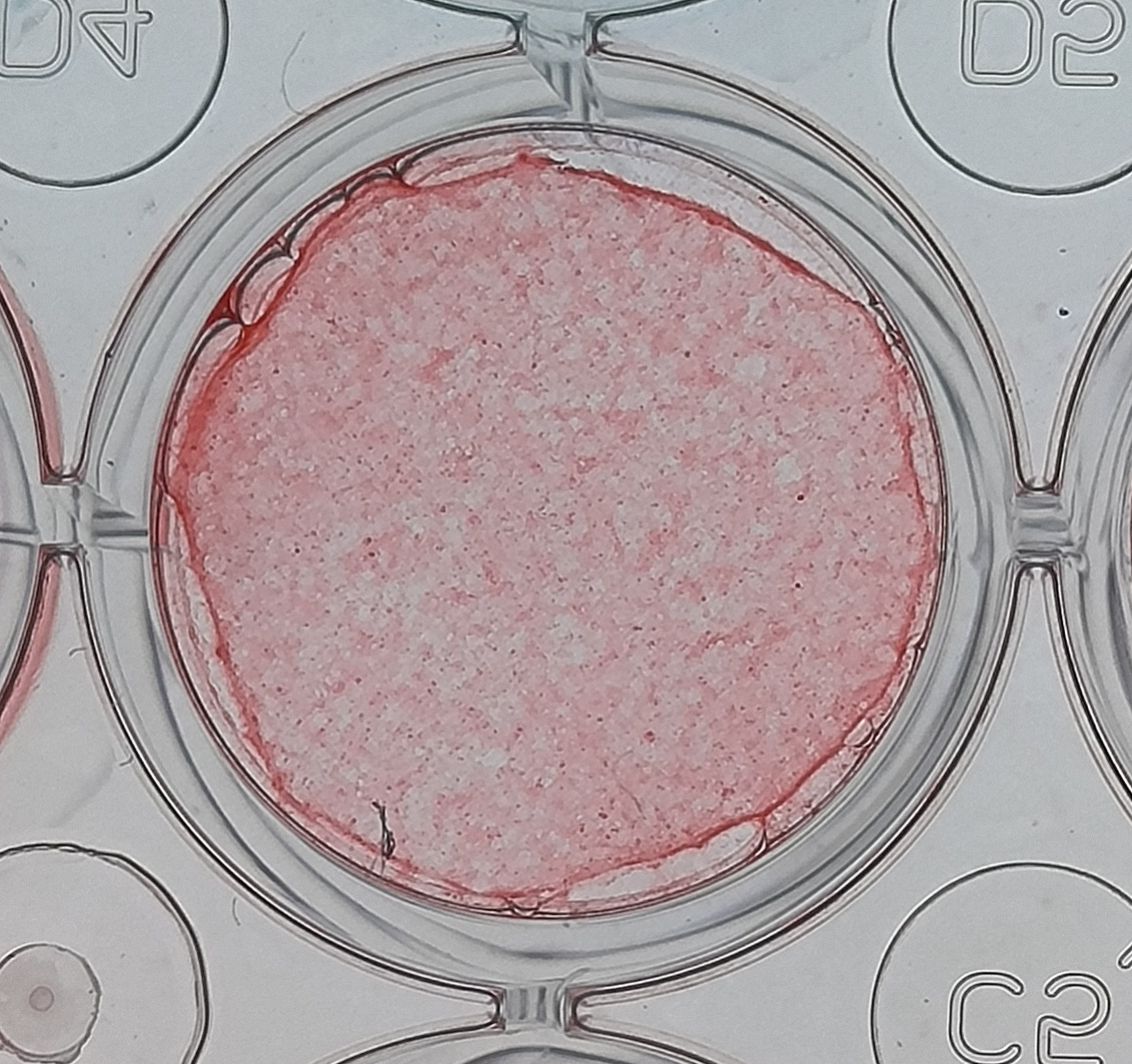

Supplement: Supplementary file 8 — Source data Fig. 5 [file 44318_2025_399_MOESM8_ESM.zip › Figure 5/5E/Figure5E-Mettl3KO+saline-whole.tif]

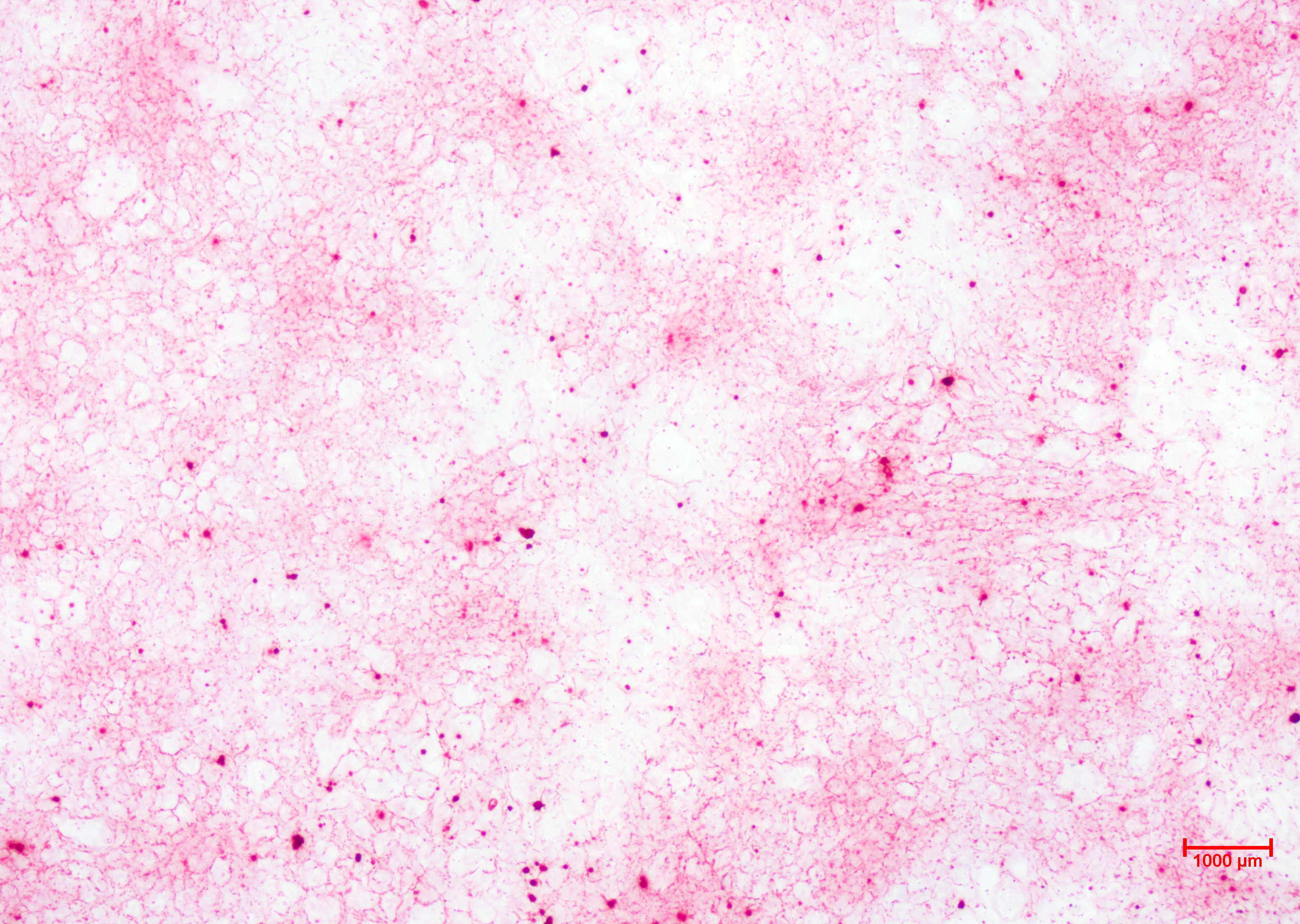

Supplement: Supplementary file 8 — Source data Fig. 5 [file 44318_2025_399_MOESM8_ESM.zip › Figure 5/5E/Figure5E-Mettl3KO+saline.tif]

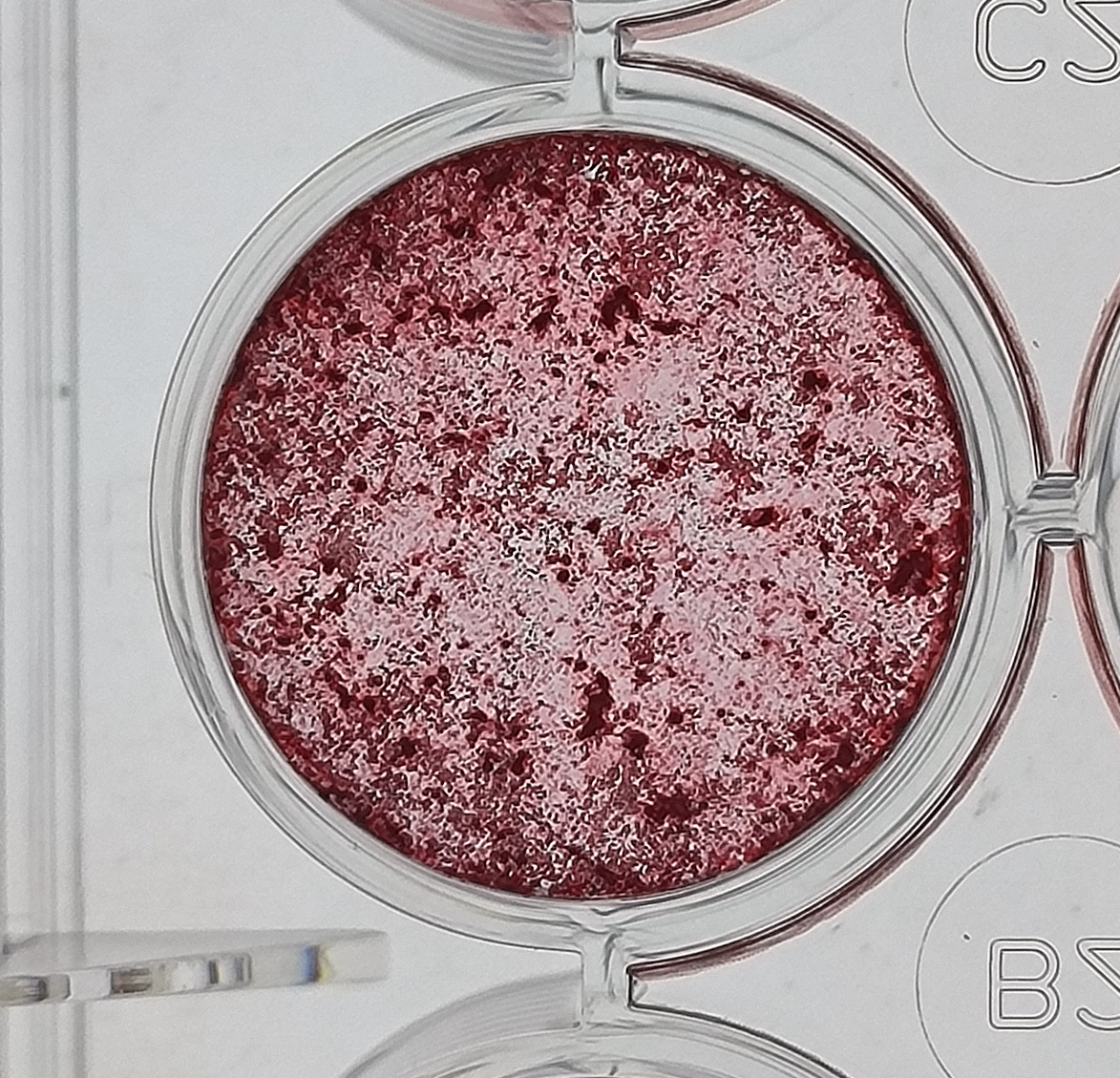

Supplement: Supplementary file 8 — Source data Fig. 5 [file 44318_2025_399_MOESM8_ESM.zip › Figure 5/5E/Figure5E-WT+saline-whole.tif]

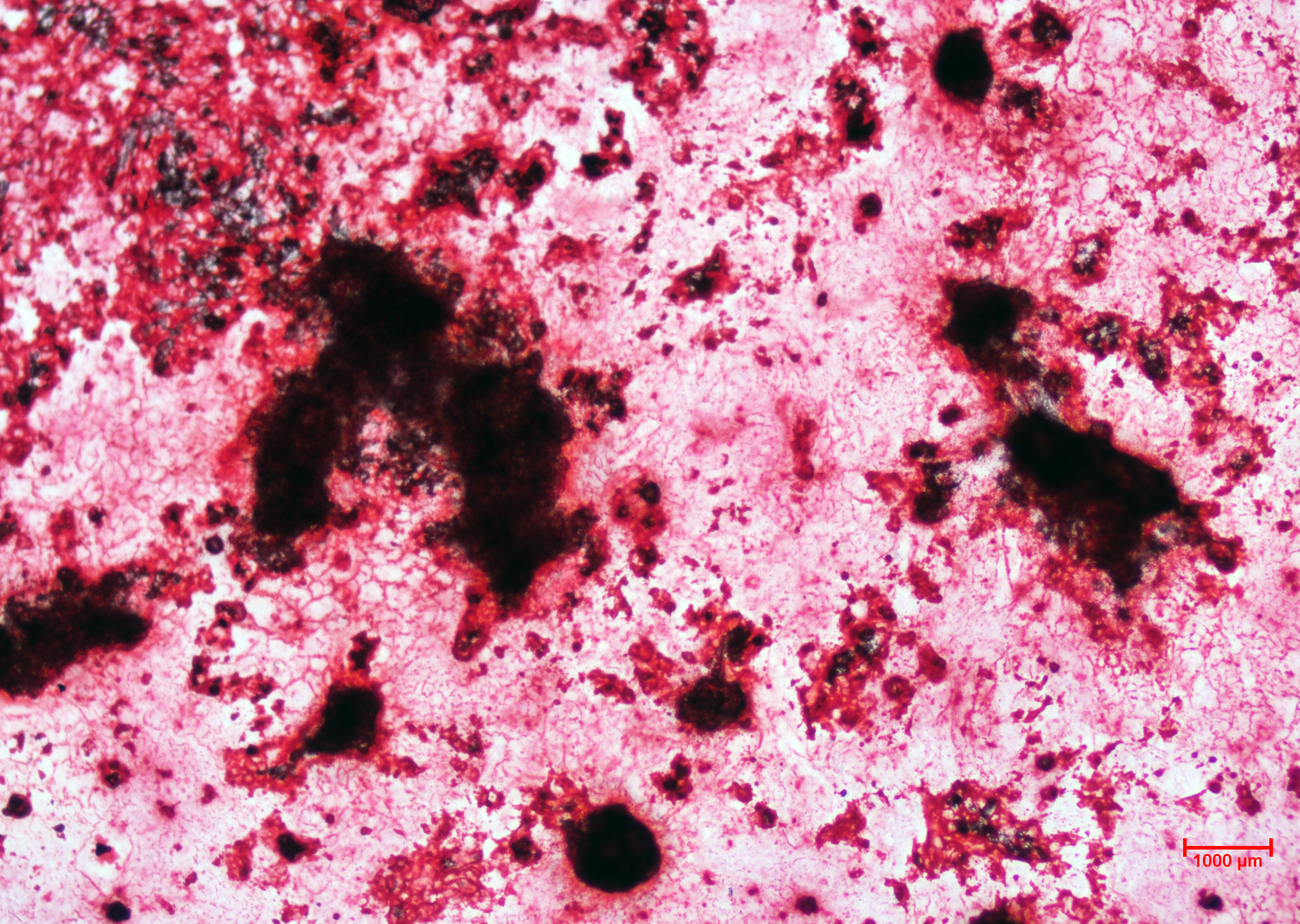

Supplement: Supplementary file 8 — Source data Fig. 5 [file 44318_2025_399_MOESM8_ESM.zip › Figure 5/5E/Figure5E-WT+saline.tif]
